# Impacts of plant growth promoters and plant growth regulators on rainfed agriculture

**DOI:** 10.1371/journal.pone.0231426

**Published:** 2020-04-09

**Authors:** Naeem Khan, Asghari M. D. Bano, Ali Babar

**Affiliations:** 1 Department of Agronomy, Institute of Food and Agricultural Sciences, University of Florida, Gainesville, Florida, United States of America; 2 Department of Bioscinces, University of Wah, Wah Cantt, Pakistan; Hainan University, CHINA

## Abstract

Demand for agricultural crop continues to escalate in response to increasing population and damage of prime cropland for cultivation. Research interest is diverted to utilize soils with marginal plant production. Moisture stress has negative impact on crop growth and productivity. The plant growth promoting rhizobacteria (PGPR) and plant growth regulators (PGR) are vital for plant developmental process under moisture stress. The current study was carried out to investigate the effect of PGPR and PGRs (Salicylic acid and Putrescine) on the physiological activities of chickpea grown in sandy soil. The bacterial isolates were characterized based on biochemical characters including Gram-staining, P-solubilisation, antibacterial and antifungal activities and catalases and oxidases activities and were also screened for the production of indole-3-acetic acid (IAA), hydrogen cyanide (HCN) and ammonia (NH_3_). The bacterial strains were identified as *Bacillus subtilis*, *Bacillus thuringiensis* and *Bacillus megaterium* based on the results of *16S-rRNA* gene sequencing. Chickpea seeds of two varieties (Punjab Noor-2009 and 93127) differing in sensitivity to drought were soaked for 3 h before sowing in fresh grown cultures of isolates. Both the PGRs were applied (150 mg/L), as foliar spray on 20 days old seedlings of chickpea. Moisture stress significantly reduced the physiological parameters but the inoculation of PGPR and PGR treatment effectively ameliorated the adverse effects of moisture stress. The result showed that chickpea plants treated with PGPR and PGR significantly enhanced the chlorophyll, protein and sugar contents. Shoot and root fresh (81%) and dry weights (77%) were also enhanced significantly in the treated plants. Leaf proline content, lipid peroxidation and antioxidant enzymes (CAT, APOX, POD and SOD) were increased in reaction to drought stress but decreased due to PGPR. The plant height (61%), grain weight (41%), number of nodules (78%) and pod (88%), plant yield (76%), pod weight (53%) and total biomass (54%) were higher in PGPR and PGR treated chickpea plants grown in sandy soil. It is concluded from the present study that the integrative use of PGPR and PGRs is a promising method and eco-friendly strategy for increasing drought tolerance in crop plants.

## Introduction

Change in current climate resulted change in temperature and precipitation profiles, leading to intense drought condition. These fluctuation in ecological condition resulted an increase in global warming which in turn resulted an increase in demand for irrigation [1]. On the other hand rise in population resulted severe devastation of prime cropland due to increase in soil erosion and urbanization. Therefore, there is an inordinate need to utilize soils with minimal ability for crop growth and production [2]. Sandy soils are poorer in plant growth and have less ability for water passage from deeper layers of soils through capillary transport. These soils have loose structure and light in structure due to which they drain very quickly. However, the fertility status and yield capabilities of these soils can be improved with the application of compost leaf mould, manure or by the application of PGPR [3]. There is an increasing demand for improving tolerance in pulses especially in chickpea against drought, in order to fulfil the world food demand [4]. Thus, policies may be develop to accomplish crops plants against drought stress and to develop drought tolerance in crop plants [5].

Bacteria that live in the locality of plant roots and interact with plants and enhance their growth directly or indirectly are known as plant growth-promoting rhizobacteria (PGPR). PGPR improve the plant growth and increase their yield as they improve the root growth and thus enhance the accessibility of micro-nutrients to the roots of host plant [6]. Plant roots produces an array of organic compounds that secrete form the roots as exudates and attract soil microbes including PGPR, as they are efficient source of carbon inside soil [7, 8]. Soil bacteria, maintain mutualistic interactions with plant roots that enable plants to grow well and tolerate several abiotic stresses [9, 10]. Rhizobacteria service plants to preserve an encouraging water status under water deficit condition by improving the growth of the root system [11]. Plant roots also perform an imperative role in water use efficiency (WUE) and PGPR further augment the water absorption ability of roots under water scarcity [12]. Inoculation of plants with PGPR results an increase in growth rate, seedling emergence, and improve the responses of plants to various stimuli and plant pathogen. PGPR induce increase in the development and yield of crop has been confirmed in both green house and field trials [13, 14]. They were also stimulatory to the growth and yield of rice, radish, sugar beet, potato, apple, tomato, wheat, beans and in ornamental plants [15, 16]. Many mechanisms have been described for the action of PGPR [17]. Some of them produces different types of plant metabolites such as hydrogen cyanide (HCN), 2,4-diacetylphloroglucinol (DAPG) [18]; antibiotics, e.g. phenazine [19]; and volatile compounds that motivate plant growth [20]. Other strains produce siderophores, biofilm and plant hormones which influence plant physiological processes [21].

Plant growth regulators also perform a significant role in plant developmental process and thus modulate plant replies to abiotic stresses. They have been found to improve the damages caused by abiotic stresses. Salicylic acid (SA) is known for its defensive role when present in plants under appropriate concentration [22–23]. SA was shown to be responsible for drought tolerance in plants [24]. Foliar spray of SA repairs the negative effects of drought and increases the restoration process in plants [25–27]. Putrescine also play constructive role in decreasing the opposing effects of abiotic stresses on plants as it has acid neutralizing and cell wall stabilizing abilities [28]. Putrescine has the ability to develop tolerance in plants against drought, oxidative and salinity stress [29, 30] and also control plant developmental process [31]. The present study was therefore aimed to evaluate the effects of bacterial isolates, salicylic acid and putrescine alone or in combination on secondary metabolites, growth and yield of chickpea grown under sandy soil conditions.

## Materials and methods

The experiments were carried out using chickpea plants grown under natural condition of field. The experiments were performed during the chickpea growing seasons 2014–15 and 2015–16. Seeds were grown in the sandy soil at Girot (soil moisture 6%), 20 km away from Khushab. Khushab is the driest and hot district with varied topographical condition, having arid hills of salt range with bushy vegetation in its north (soon sakesar valley) and central part have irrigated low land plains and southern part has hot dry desert with scarce vegetation. The temperature ranges from 24–50 ^o^C in summer and 20–30 ^o^C in winter with normal yearly precipitation of 370 millimetre. Seeds of two chickpea varieties i.e., Punjab Noor-2009 (drought sensitive) (Shah et al. 2016) and 93127 (drought tolerant) (Irshad et al. 2010), were obtained from Ayub Agriculture Research Institute, Faisalabad. Bacterial colonies were secluded from the rhizosphere of chickpea plants grown in sandy soil of Karak, Bhakkar and Cholistan (with 7%, 6% and 4% soil moisture contents) and were named as P_1_, P_2_ and P_3_. The experiment was carried out in a Randomized Complete Block Design (RCBD) with a plot size of 5 ×1. 5 m, with four replications.

The experiment had 11 treatments which are described below

T_1-_ Seeds inoculated with *Bacillus subtilis*

T_2-_ Treatment with *Bacillus subtilis* + 2 PGRs

T_3-_ Inoculation of seeds with *Bacillus subtilis and Bacillus thuringiensis*

T_4-_ Inoculation of seed with *Bacillus subtilis* and *Bacillus thuringiensis* + Plants Sprayed with both the PGRs

T_5-_ Seeds inoculated with *Bacillus subtilis*, *Bacillus thuringiensis* and *Bacillus megaterium*.

T_6-_ Combined treatment of all 3 PGPR and 2 PGRs

T_7-_ Plants treated with SA

T_8-_ Plants treated with Put

T_9-_ combined treatment of SA and Put

T_10-_ Untreated control

T_11-_ Irrigated control

### Collection of soil samples

Soil samples were collected at 6 inches from top soil from three rain-fed areas (Karak, Bhakkar and Cholistan) of Pakistan, with 7%, 6% and 4% of soil moisture contents. The method of McKeague [32] and McLean [33] was followed for determination of soil pH and electrical conductivity (EC).

### Isolation and purification of PGPR strains

Bacterial strains were isolated from the rhizosphere of chickpea. Decimal dilutions were made from the supernatant of all soil samples and were spread (20 μl) on Luria-Bertani (LB) agar plates. The agar plates were incubated for 2 days. The appeared bacterial colonies agar plates were streaked 6–7 times till purification.

### Sterilization of seeds

Before seed inoculation, they were sterilized with ethanol (70%) and clorox (10%) for 3 minutes and washed with autoclaved distilled water [34].

### Seed inoculation with bacterial culture

The inoculated Luria Bertani (LB) broth was used for seeds inoculation before sowing.

### Characterization of bacterial isolates for beneficial plant growth promoting traits

#### Morphology and colony of isolated PGPR

Bacterial isolates were grown on pikovskaya’s overnight and the isolates were placed on agar plates [35]. The color and shape of the colonies was recorded after 24 hours.

### Gram staining

For gram staining, slides of bacterial strains were prepared, following the method of Vincent [36].

### Oxidase and catalase test

The oxidase tests was performed following the method adopted by Steel [37] while, for the determination of oxidase activity, kovacs reagent [38] was used.

### IAA production by selected PGPR strains

Indole-3-acetic acid (IAA) production by selected PGPR was determined by a colorimetric method using the Salkowski's reagent [39]. The optical density was recorded at 530 nm. IAA production was matched with YMD and LB media and YMD medium was also matched with and without tryptophan.

### Hydrogen Cyanide (HCN) production by selected PGPR strains

Selected strains were screened for hydrogen cyanide production following the method of Lorck [40]. The whatman No. 1 filter paper was used for this purpose and change in filter paper color from yellow to light brown, brown or reddish brown was recorded for weak (+), moderate (++) and strong (+++) reaction respectively.

For quantitative analysis of HCN, bacterial cultures were grown in used King’s B broth augmented with glycine (4.4 g/ l) and its absorbance was read at 625 nm [41].

### Ammonia (NH_3_) production by selected PGPR strains

The method of Cappuccino and Sherman [42] was adopted for NH_3_ production. The presence of NH_3_ was shown by alteration in color from yellow to brown.

### Phosphate Solubization Index (PSI)

For PSI Pikovskaya’s media was inoculated with bacterial isolates and were incubated for 7 days (28°C). Solubilization index (SI) was determined by using the formula of Pikovskaya [34].

SI = diameter (cm) + halozone (cm)/ diameter (cm)

### Extraction of bacterial DNA

A single colony of bacterial culture was used to inoculate tryptone yeast extract (TY) broth. The inoculated TY broth was incubated overnight in a shaker (Model: Excella E-24). The centrifugation was done twice by adding with 100% ethanol to clean the obtained DNA. The DNA was dissolved in distilled water. The purity of DNA was assessed through nanodrop spectrophotometry (260–280 nm) [43].

### PCR-amplification and *16S rRNA* sequence analysis

Weisburg et al. [44] method was used for amplification of genomic DNA. The obtained amplified PCR products were electrophoresed (1.2% w/v) agarose gel with a DNA ladder of 1 kb as molecular marker. Ethidium bromide (0.01 gm/ml) was applied to the gel and examined under UV trans-illuminator lamp. Approximately 1400 bp purified PCR products were sequenced by using primers 27FAgAgTTTgATCMTGGCTCAg, 1492RTACggYTACCTTgTTACgACTT, 518FCCA gCAgCCgCggTA ATA Cg, and 800R TAC CAgggT ATC TAA TCC.

### Biochemical analyses of crop plants

#### Leaf chlorophyll content

The soil plant analysis development (SPAD) chlorophyll meter was used for the determination of chlorophyll content of plant leaves, Chlorophyll content of three leaves in each plant was measured.

#### Leaf proline content

Bates *et al*. [45] method was used for the estimation of proline content of leaves. The absorbance of upper layer of the solution was recorded at 520 nm and total proline was calculated as:

Proline μg/g = k value x dilution factor x absorbance/fresh sample wt.

K value = 17.52, Dilution factor = 2, Wt. of sample = 0.5 g

### Leaf protein content

Lowery *et al*. [46] method was followed for the determination of protein content in the leaves of crop plants using BSA (Bovine Serum Albumen) as standard. The absorbance of each sample was determined at 650 nm along with the absorbance of different concentrations of bovine serum albumen (BSA). Protein concentration was calculated by using the below mentioned formula:

Protein content mg/g = K value × Dilution factor × Absorbance/sample wt.

K value = 19.6, Dilution factor = 2, Wt. of sample = 0.1 g

### Sugar estimation

Sugar content was estimated, following the method of Dubois *et al*. [47]. The concentration of sugar in unknown sample was considered with reference to standard curve made from glucose:

Sugar content (mg/g) = K value × Dilution factor × Absorbance/Sample wt.

K value = 20, Dilution factor = 10, Weight of sample = 0.5 g.

### Lipid peroxidation and total phenolic content

For determination of lipid peroxidation, the amount of malondialdehyde (MDA) formed by thiobarbituric acid (TBA) reaction was calculated as defined by Li [48] whereas, folin-Ciocalteu colorimetric method [49] was used for the estimation of total phenolic content.

### Shoot fresh and dry weights

The fresh weight of five plants were measured with the help of an electronic balance. The shoots of these plants were than oven dried at 70 ^o^C and their dry weight was measured.

### Root fresh and dry weights

The roots of five plants were weighed with the help of an electronic balance for measuring their fresh weights. The selected roots were then oven dried at 70 ^o^C till constant weight for the determination of their dry weights.

### Relative Water Content (RWC)

The Relative Water Content (RWC) of each leaf was calculated according to the formula of Weatherly [50].

RWC = [(fresh mass—dry mass)/ (saturated mass—dry mass)] × 100.

### Antioxidant enzymes extraction

For determination of antioxidant enzymes activity, 0.5 g of leaves was crushed in 5 ml of 50 mM phosphate buffer while keeping on ice bath. The homogenate obtained after the procedure was centrifuged for 22 minutes at 13000 g at 4 ^o^C. The supernatant obtained was used to study the antioxidant enzymes activity including POD [51–52], APOX [53], CAT [54] and SOD [55].

### Yield and yield related parameters

#### Plant height

Five plants/replication was randomly selected and their height was recorded (in cm) at maturity from ground level to the base of the spike with the help of a meter rod.

#### Spike length

Five spikes were randomly selected in each treatment and length was measured (cm) in selected spikes from base to the tip of the spike with the help of a meter rod. The average length of 5 spikes was used for statistical analysis.

#### 100-Grain weight

Hundred grains were counted after harvest and weighed for each replication. The mean value of four replication was used in figure.

#### 100-Pod weight

Hundred pod were counted after harvest and weighed for each replication. The mean value of four replication was used in figure.

#### Total biomass

Dry weight of 20-plants was measured (in g) at maturity for each replication and used for statistical analysis.

#### Harvest index

Ratio between grain yield per 5-plants and biomass per 5-plants was calculated by using the following formula:

HI (%) = (Grain yield per 5-plants / biomass per 5-plants) × 100.

#### Data analysis

Experiments were repeated four times. The data analysis was carried by using software Statistics, version. 8.1. An ANOVA was performed to determine the effect of treatments and error associated with the experiment. To identify significant differences among treatments, a mean comparison of traits was carried out by using protected LSD (*P = 0*.*05*) test where error mean square was used to estimate the standard error of differences between mean.

## Results

The experiment was conducted in two consective vegetation season (2014–15 and 2015–16). The patteren of response of grwoth parameters to various treatments was almost simillar during both growing years but the % increase in comparison to untreated uninocualted control was greater during second year (2015–16). All the treatments exhibited substantial affects over all the studied parameters.

### Morphological and biochemical characteristics of isolated PGPR

All the isolated PGPR strains were categorised on the basis of their colony shape, cell motility, gram staining and oxidase and catalase activity. The selected strains were checked for P-solubilisation, antibacterial and antifungal activities, proline, IAA, HCN and ammonia production. All the PGPR strains were gram positive and were predominantly rod shaped, with colony color varied from white to off-white. All the isolates were found positive for oxidase and catalase activity (Table 1).

**Table 1:** Morphological, physiological and biochemical characteristics of isolated bacterial strains.

_S.NO_	_Reaction_	_Test_	_*Bacillus Megaterium*_	_*Bacillus Thuringiensis*_	_*Bacillus subtilis*_
_1_	_CS_	_COLONY SHAPE_	_Rod_	_Irregular_	_Rod_
_2_	_CM_	_CELL MOTILITY_	_Motile_	_Motile_	_Motile_
_3_	_GS_	_GRAM STAINING_	_+_	_+_	_+_
_4_	_OXID_	_OXIDASE_	_+_	_+_	_+_
_5_	_CAT_	_CATALASE_	_+_	_+_	_+_
_6_	_ONPG_	_ORTHO NITRO PHENYL GALACTOPYRANOSIDE_	_+_	_+_	_+_
_7_	_CIT_	_SODIUM CITRATE_	_-_	_+_	_-_
_8_	_MALO_	_SODIUM MELONATE_	_-_	_-_	_+_
_9_	_LDC_	_LYSINE DECASE_	_+_	_+_	_+_
_10_	_ADH_	_ARGININE DIHYDROLASE_	_-_	_-_	_-_
_11_	_ODC_	_ORNITHINE DECARBOXYLASE_	_-_	_+_	_+_
_12_	_H2S_	_H2S PRODUCTION_	_-_	_+_	_+_
_13_	_UREA_	_UREA HYDROLYSIS_	_+_	_+_	_-_
_14_	_TDA_	_TRYPHTOPHANE DEAMINASE_	_+_	_-_	_-_
_15_	_IND_	_INDOLE_	_-_	_+_	_+_
_16_	_VP_	_VOGES PROSKAUER_	_-_	_-_	_-_
_17_	_GEL_	_GELATIN HYDROLYSIS_	_+_	_-_	_-_
_18_	_GLU_	_ACID FROM GLUCOSE_	_+_	_+_	_+_
_19_	_NO3/N2_		_+_	_+_	_+_
_20_	_MALT_	_ACID FROM MALTOSE_	_+_	_+_	
_21_	_SUC_	_ACID FROM SUCROSE_	_+_	_+_	_+_
_22_	_MANN_	_ACID FROM MANNOSE_	_-_	_-_	_+_
_23_	_ARAB_	_ACID FROM ARABINOSE_	_+_	_-_	_+_
_24_	_RHAM_	_ACID FROM RHAMNOSE_	_+_	_+_	
_25_	_SORB_	_ACID FROM SORBITOL_	_-_	_-_	
_26_	_INOS_	_ACID FROM INOSITOL_	_-_	_+_	
_27_	_ADO_	_ACID FROM ADONITOL_	_-_	_-_	
_28_	_MEL_	_ACID FROM MELIBIOSE_	_+_	_+_	
_29_	_RAF_	_ACID FROM RAFFINOSE_	_-_	_-_	

+ Present,—Absent

### Phosphorus Solubilisation Index (PSI)

The three isolated PGPR strains *i*.*e*. *Bacillus subtilis*, *Bacillus thuringiensis* and *Bacillus megaterium* were phosphate solubilizers (Table 2). *Bacillus subtilis* was with the greatest potential for phosphorus solubilization with PSI of 2.822. The PSI for *Bacillus megaterium* and *Bacillus thuringiensis* were 2.621 and 2.411 respectively.

**Table 2:** P-solubilizing index of selected PGPR strains.

S.No	Isolates	Halozone diameter (mm)	P-solubilisation index
1	*Bacillus subtilis*	1.4	2.822
2	*Bacillus thuringiensis*	1.1	2.411
3	*Bacillus megaterium*	1.3	2.621

### Proline, IAA, HCN and NH_3_ production by selected PGPR isolates

Maximum proline production (1.699 ug/mg) was recorded in *Bacillus thuringiensis* followed by *Bacillus megaterium* (1.671 ug/mg) whereas, *Bacillus subtilis* was more effective in producing indole 3-acetic acid (Table 3). All the 3-selected PGPR isolates were tested for the production of hydrogen cyanide and found that all of them (except *B*. *subtilis*) were adept to change the color of filter paper from yellow to orange or dark brown which indicated the presence of hydrogen cyanide. In quantitative analysis, *Bacillus megaterium* was found most effective with maximum O.D value of 0.097 followed by *Bacillus thuringiensis* (0.082), for hydrogen cyanide production. Similarly, all the strains were found positive for ammonia production.

**Table 3:** Proline, IAA, HCN production by Selected PGPR strains and detection of NH_3_.

S.No	Selected PGPR Strains	Proline Production (μg/mg)	IAA Production (μg/ml)	HCN production		NH_3_ Detection
				Qualitative	Quantitative (OD readings)	
1	*B*. *subtilis*	1.011	0.499	-	0.011	+
2	*B*. *thuringiensis*	1.699	0.442	+++	0.082	+
3	*B*. *megaterium*	1.671	0.381	+++	0.097	+

HCN production (based on intensity of color):—negative, +weak, ++ moderate, +++ strong

### Alignment of *16S rRNA* sequence

For the isolate P_1_, isolated from the rhizosphere of chickpea (at Karak, 7% soil moisture content), a total length of sequence with 1557 nucleotid was obtained. The evaluation of the nucleotide sequence with data nucleotide bank indicated 100% (1506/1506) similarity with *Bacillus subtilis* (Accession No. MF616407). For the isolate P_2_, obtained from the rhizosphere of chickpea (at Bhakkar, 6% soil moisture content), the total length of sequence with 1517 nucleotide was obtained. The evaluation of the nucleotide sequence with data nucleotide bank indicated sequence similarity of 99% (1514/1517) with *Bacillus thuringiensis* (Accession No. MF662971). For the isolate P_3_, isolated from the rhizosphere of chickpea (at Cholistan, 4% soil moisture content), the total length of sequence with 1474 nucleotide was obtained. The evaluation of the nucleotide sequence with data nucleotide bank showed maximum sequence similarity of 99% (1492/1498 bases) with *Bacillus megaterium* (Accession No. MF008110).

### Biochemical characters

#### Chlorophyll content

**Fig 1:**
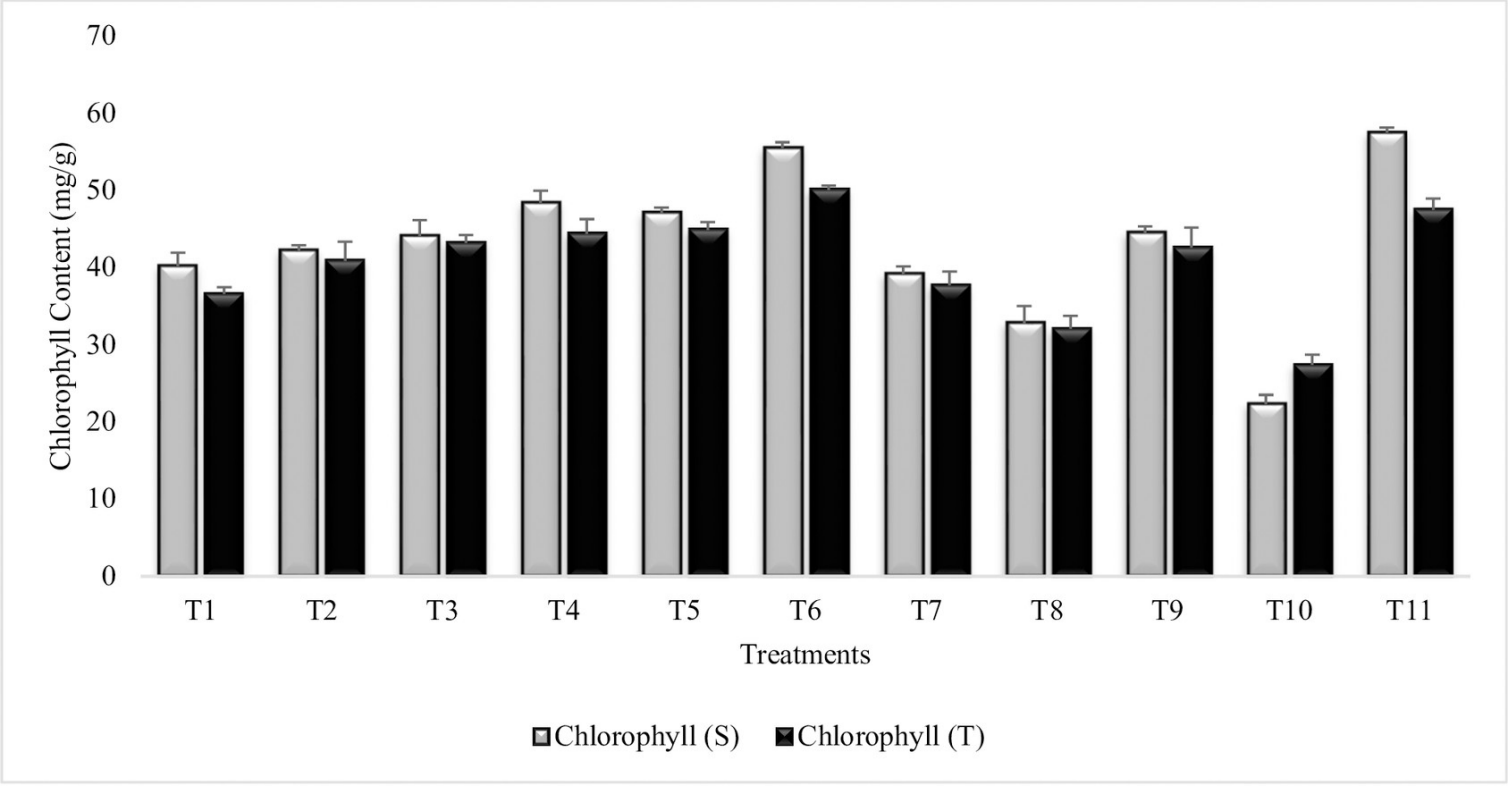
Chlorophyll content of chickpea grown in sandy soil. Data are means of four replicates along with standard error bars (S-Sensitive Variety; T-Tolerant Variety). T_1_- Seeds inoculated with P_1_, T_2_- Seeds inoculated with P_1_ + Plants Sprayed with SA and Put, T_3_- Seeds inoculated with P_2_ and P_3_, T_4_- Seeds inoculated with P_2_ and P_3_+ Plants Sprayed with SA and Put, T_5_- Seeds inoculated with P_1_, P_2_ and P_3_, T_6_- Seeds inoculated with P_1_, P_2_ and P_3_ + Plants Sprayed with SA and Put, T_7_- Plants sprayed with SA, T_8_- Plants sprayed with Put, T_9_- Plants sprayed with SA and Put, T_10_- Untreated control plants grown in sandy soil, T_11_- Irrigated control plants.

In comparison to untreated control plants grown in sandy soil (T_10_) chlorophyll content was improved in all the treatments in both the varieties (Fig 1). Maximum increase (59% and 45%) was noted in T_6_ (*B*. *subtilis*, *B*. *thuringiensis* and *B*. *megaterium* in combination with PGRs), and the increase in T_6_ was greater than irrigated control (T_11_) for tolerant variety. The sensitive variety had higher chlorophyll content then tolerant variety in all treatments except for stress control (T_10_). The least increase was recorded in Put treatment. Combined treatment of both PGRs (T_9_) was more effective than SA (T_7_) and Put (T_8_) alone. The performance of PGPR was higher than PGR particularly the Put treatment. Similar results were recorded for all treatments during succeeding year 2015–16 (S1 Table).

#### Leaf proline content

**Fig 2:**
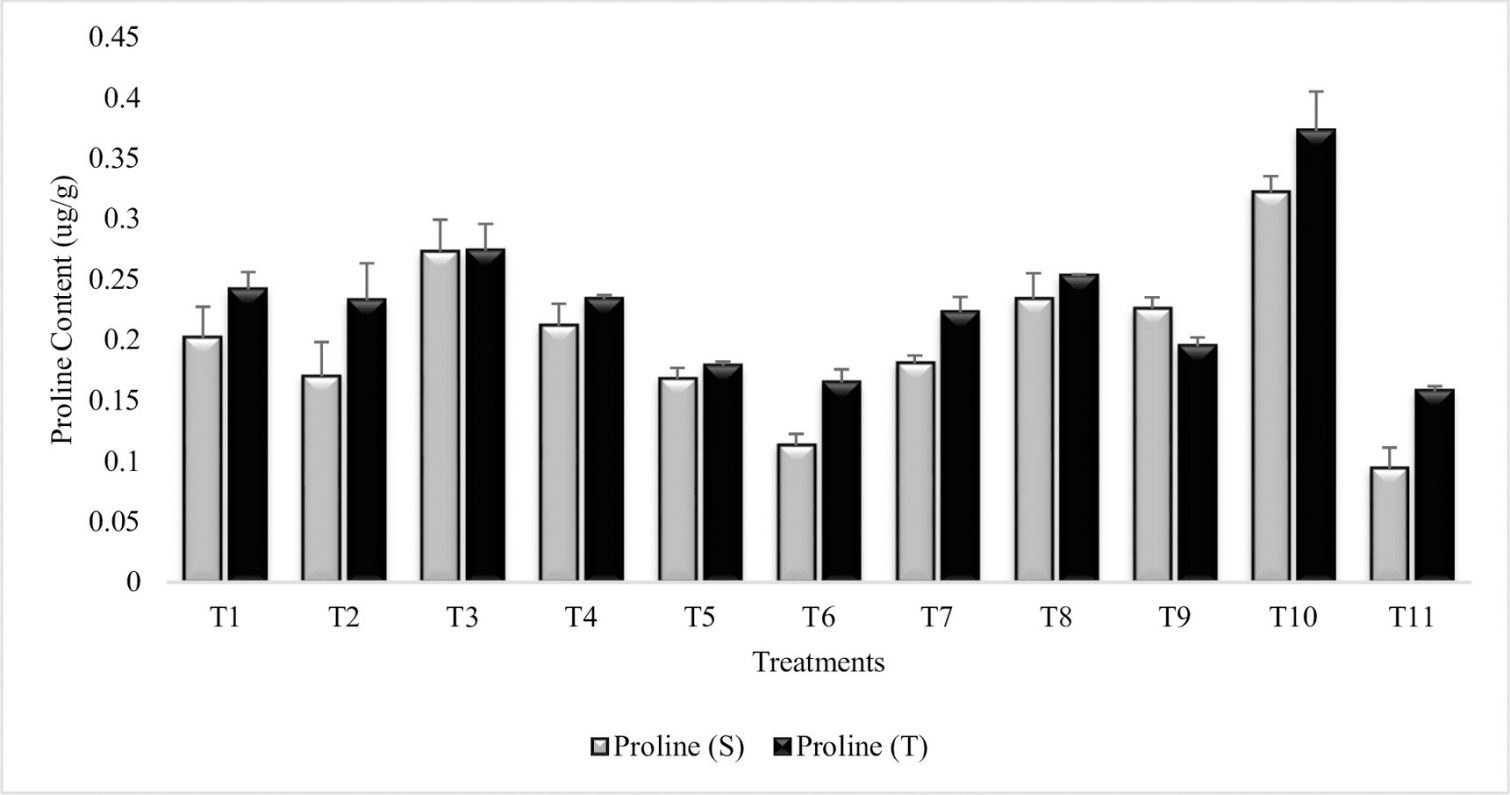
Proline content of chickpea grown in sandy soil. Data are means of four replicates along with standard error bars (S-Sensitive Variety; T-Tolerant Variety).

The result revealed significant decrease in leaf proline content as compared to stress control (T_10_), though the values were greater than irrigated control (T_11_) (Fig 2). T_6_ (Combined treatment of *B*. *subtilis*, *B*. *thuringiensis* and *B*. *megaterium* in combination with PGRs) had equally decreased the leaf proline content as compared to irrigated control in tolerant variety. The tolerant variety displayed enhanced proline accumulation over sensitive variety in most treatments. Treatment with *B*. *subtilis* and *B*. *thuringiensis* (T_3_) was at par in both the varieties and was least effective for decreasing proline content among all the treatments. The combined treatment of plant growth regulators (T_9_) was more effective in tolerant variety for the decrease in leaf proline content whereas, SA (T_7_) and Put (T_8_) resulted less proline accumulation in sensitive variety. Similar pattern of decrease in leaf proline content was recorded in the 2^nd^ year (S2 Table).

#### Leaf protein content

**Fig 3:**
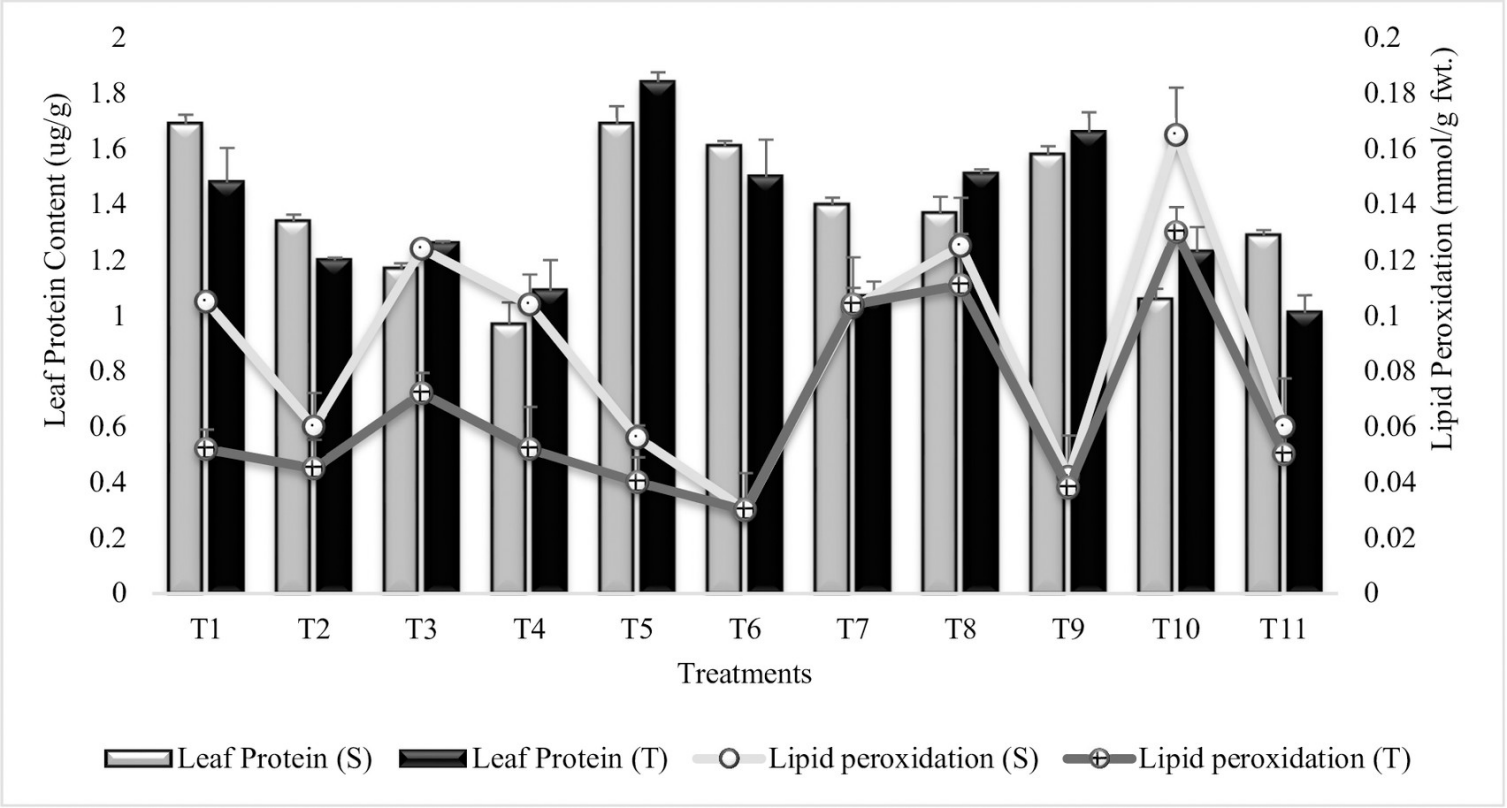
Leaf protein and lipid peroxidation contents of chickpea grown in sandy soil. Data are means of four replicates along with standard error bars (S-Sensitive Variety; T-Tolerant Variety).

The result showed that T_5_ (Combined treatment of *B*. *subtilis*, *B*. *thuringiensis* and *B*. *megaterium*) had significantly (33% and 37%) enhanced the leaf protein content over uninoculated untreated plants grown in sandy soil (T_10_), followed by T_1_ (inoculation with *Bacillus subtilis*) in both the varieties. T_1_, T_5_, T_6_, T_8_ and T_9_ had higher protein accumulation, even the values were greater than irrigated control (T_11_) (Fig 3). Treatments T_1_, T_6_ and T_8_ were at par, for leaf protein accumulation in tolerant variety whereas, T_1_ = T_5_ and T_6_ = T_9_ in sensitive variety. T_2_, T_4_ and T_6_ had lower values as compared to T_1_, T_3_ and T_5_, demonstrating that PGR (SA and Put) had no or little role on accumulation of leaf protein content when applied in combination with PGPR. SA (T_7_) alone was more effective (23%) in sensitive variety than tolerant variety. SA in combination with Put (T_9_) was more effective than their separate application (T_7_ and T_8_). Put was more effective (9%) than SA (T_7_) in tolerant variety but was at par in sensitive variety. Similar pattern of increase was recorded for protein content in the succeeding year 2015–16 (S3 Table).

#### Lipid peroxidation

Substantial reduction in the content of lipid peroxidation was noted in all the treatments in comparison to control plants grown in sandy soil (T_10_) (Fig 3). The highly significant decrease (81% and 76%) in lipid peroxidation was noted in T_6_ (*B*. *subtilis*, *B*. *thuringiensis* and *B*. *megaterium* in combination with SA and Put) followed by T_9_ (SA and Put) for both the sensitive and tolerant varieties. In general, the rise in lipid peroxidation was greater in sensitive variety than tolerant variety, except for T_6_ and T_7_ (SA treatment), which were at par in both the varieties. Treatments T_1_, T_4_ and T_7_ had equal % decrease in lipid peroxidation for sensitive variety. Similar results were recorded for all the treatments during succeeding year 2015–16 (S4 Table).

#### Leaf sugar content

**Fig 4:**
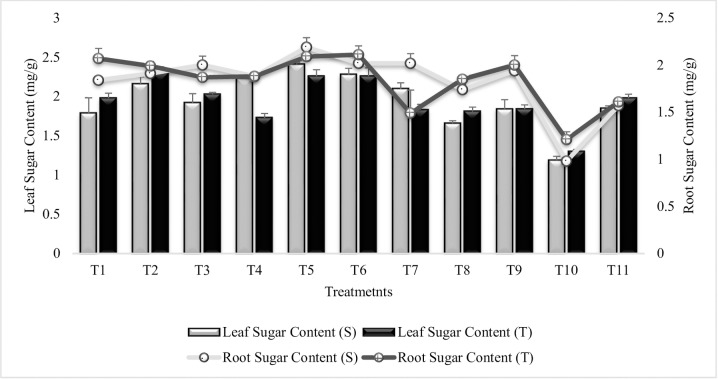
Leaf and root sugar contents of chickpea grown in sandy soil. Data are means of four replicates along with standard error bars (S-Sensitive Variety; T-Tolerant Variety).

As compared to control plants grown in sandy soil (T_10_), leaf sugar content was increased in all the treatments of both the varieties (Fig 4). Significant increase (50% and 42%) in leaf sugar content was recorded in T_5_ (Combined treatment of *B*. *subtilis*, *B*. *thuringiensis* and *B*. *megaterium*) followed by T_6_ (Combined treatment of *B*. *subtilis*, *B*. *thuringiensis* and *B*. *megaterium* in combination with SA and Put). Sensitive variety had higher values in T_4_, T_5_ and T_7_ than tolerant variety. SA (T_7_) was more effective for the increase in leaf sugar content than Put (T_8_) and combined treatment of SA and Put (T_9_). SA and Put had equal % increase in tolerant variety if applied alone or in combination. Similar results were recorded for all the treatments in the succeeding year 2015–2016 (S5 Table).

#### Root sugar content

The root sugar was lower in T_7_ (SA treatment) of tolerant variety as compared to irrigated control, all other treatments had significantly increased the root sugar content as compared to stress or irrigated control (T_11_) (Fig 4). T_5_ (Combined treatment of *B*. *subtilis*, *B*. *thuringiensis* and *B*. *megaterium*) showed maximum increase (55%) in root sugar content followed by T_6_ (Combined treatment of *B*. *subtilis*, *B*. *thuringiensis* and *B*. *megaterium* in combination with SA and Put) in sensitive variety whereas, in tolerant variety maximum increase (42%) was recorded in T_6_ followed by T_5_. T_1_, T_5_, T_6_ and T_9_ were at par in tolerant variety whereas, T_6_ = T_4_ for sensitive variety. T_4_ (Combined treatment of *B*. *thuringiensis* and *B*. *megaterium* in combination with SA and Put) had equal % increase in both the tolerant and sensitive varieties. Plant growth regulators, had no synergistic effects on root sugar content when applied in combination with PGPR however, PGR alone were more effective in enhancing root sugar content. All the treatments followed the same pattern of increase in the succeeding second year 2015–16 (S6 Table).

#### Phenolic content of leaves

**Fig 5:**
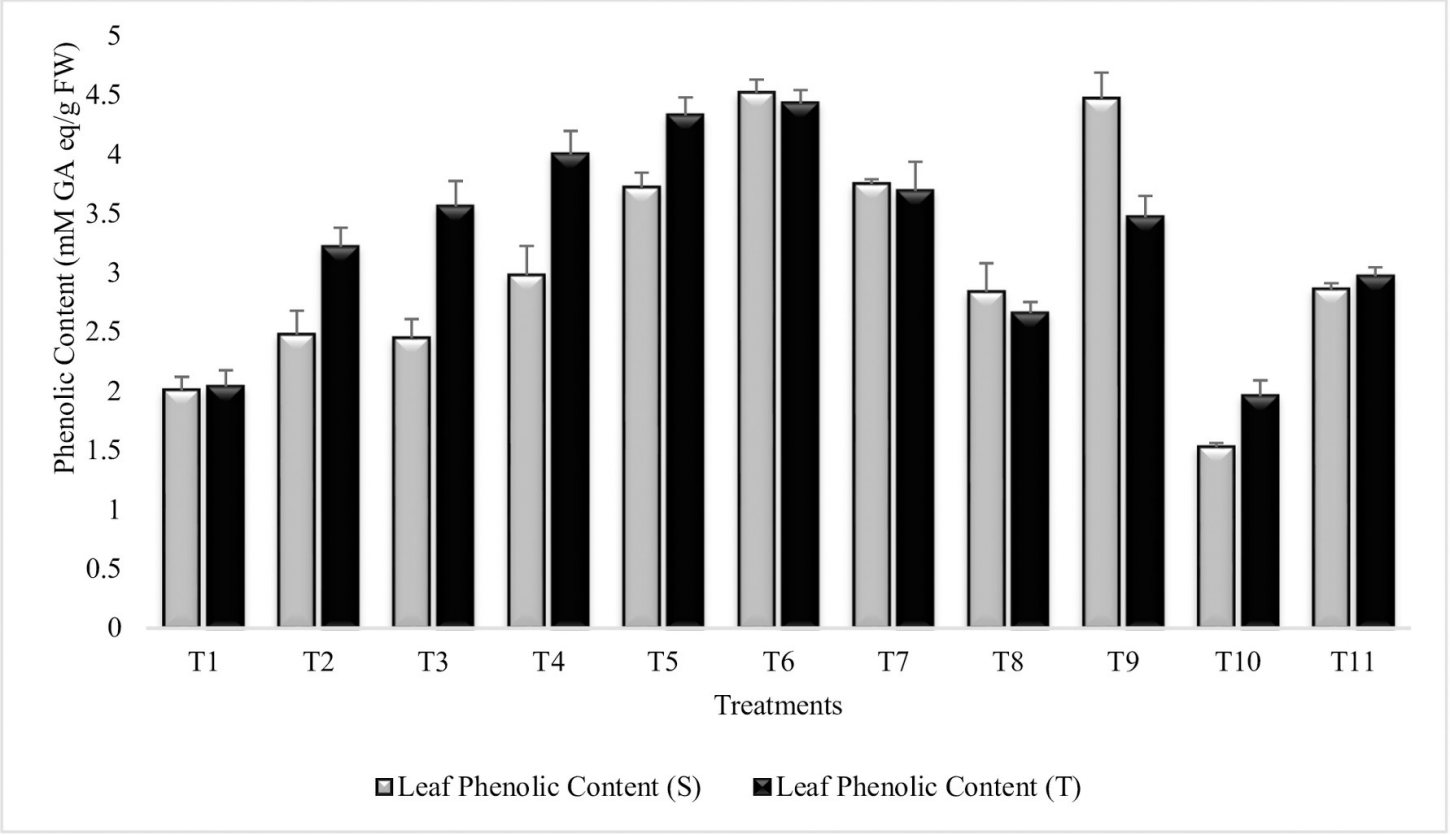
Leaf phenolic content of chickpea grown in sandy soil. Data are means of four replicates along with standard error bar (S-Sensitive Variety; T-Tolerant Variety).

The result revealed that all the treatments significantly increased the leaf phenolic content over the untreated plants grown in sandy soil (T_10_) (Fig 5). Maximum increase (66% and 55%) in phenolic content was recorded in T_6_ (Combined treatment of *B*. *subtilis*, *B*. *thuringiensis* and *B*. *megaterium* in combination with SA and Put) for both the tolerant and sensitive varieties and T_6_ = T_9_ for sensitive variety whereas, T_6_ = T_5_ for tolerant variety. Phenolic content was higher in combined treatment of SA and Put (T_9_) for sensitive variety over tolerant variety. T_3_ (treatment with *Bacillus subtilis* and *Bacillus thuringiensis*) had similar effect on leaf phenolic content of tolerant variety as compared to T_9_ (SA and Put) and T_5_ = T_7_ for sensitive variety. SA (T_7_), was more effective than Put (T_8_) in both the varieties. Combined treatment of SA and Put (T_9_), was more responsive than SA and Put alone in sensitive variety and significantly enhanced the leaf phenolic content over stressed control (T_10_) and irrigated control (T_11_). Similar findings were recorded during the succeeding year 2015–16 (S7 Table).

#### Antioxidant enzymes activity

**Fig 6:**
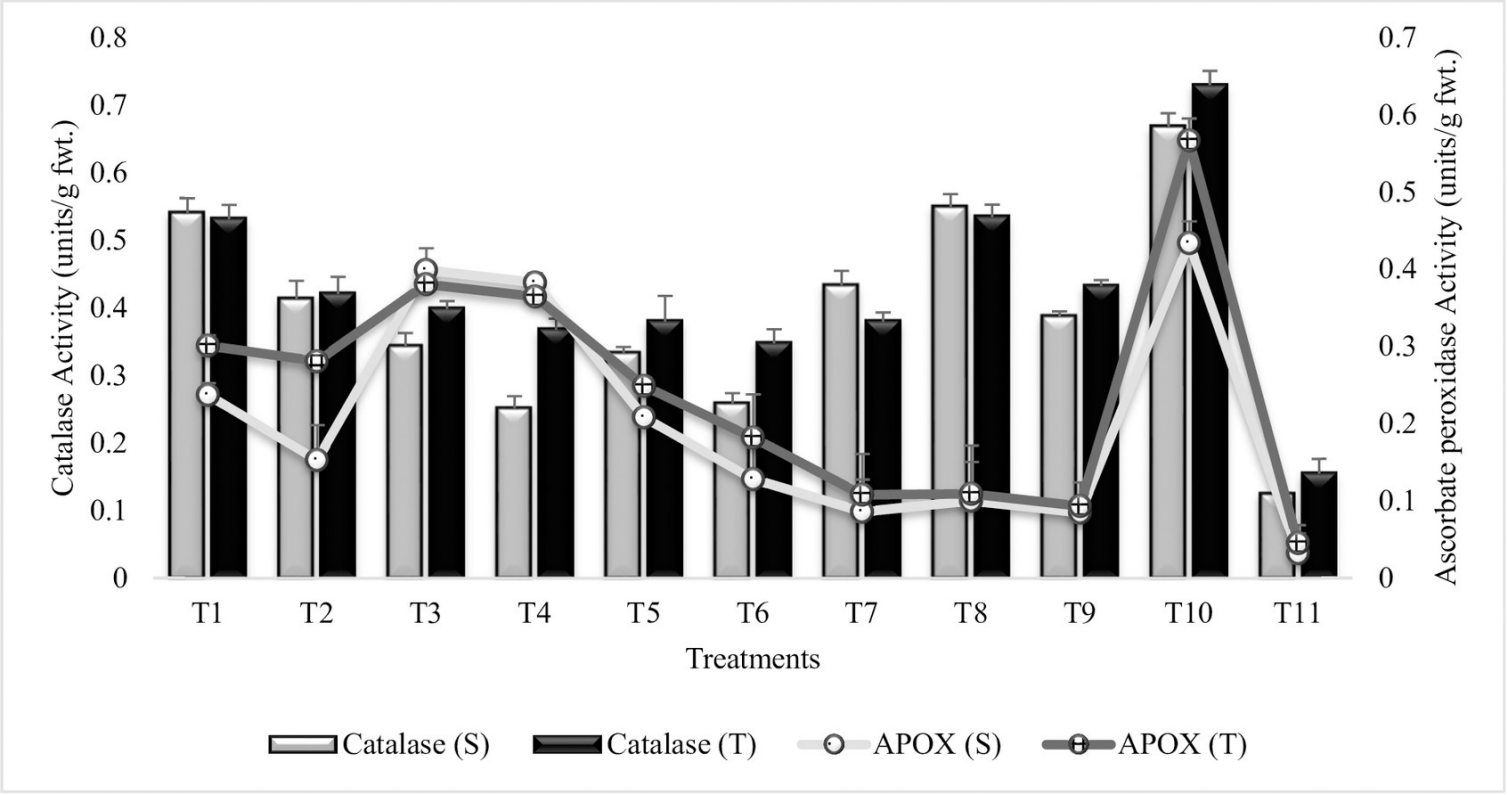
Catalase and ascorbate peroxidase activities of chickpea grown in sandy soil. Data are means of four replicates along with standard error bars (S-Sensitive Variety; T-Tolerant Variety).

All the inoculated plants showed significant decrease in catalase activity as compared to untreated plants grown in sandy soil (T_10_), though the values were higher than irrigated control (T_11_) (Fig 6). The significant reduction (64% and 40%) in catalase activity was recorded in T_4_ (combined treatment of *B*. *thuringiensis* + *B*. *megaterium* in combination with SA and Put) and T_6_ (Combined treatment of *B*. *subtilis*, *B*. *thuringiensis* and *B*. *megaterium* in combination with SA and Put) for both the sensitive and tolerant varieties. *Bacillus subtilis* (T1) was less effective in reducing catalase activity and T_1_ (treatment with *Bacillus subtilis*) and T_2_ (treatment with *Bacillus subtilis* in combination with SA and Put) had similar values for catalase activity in both the drought tolerant and sensitive varieties. The plant growth regulator, Put had equal % decrease in both the varieties whereas, SA (T_7_) was more responsive in sensitive variety. The combined treatment of SA and Put (T_9_) had similar impact on the catalase activity in T_4_ and T_6_ for sensitive variety. Similar results were reported during second year experiment (S8 Table).

Ascorbate peroxidase activity was reduced in PGPR and PGR treated plants as compared to untreated plants grown under sandy soil (T_10_) (Fig 6). Maximum reduction (80% and 83%) in ascorbate peroxidase activity was recorded in T_9_ (combined treatment of SA and Put) followed by T_7_ (SA alone) in both the sensitive and tolerant varieties, respectively. Foliar applications of SA and Put was less effective in combination with *Bacillus thuringiensis* and *Bacillus megaterium* (T_4_). Tolerant variety had higher values for ascorbate peroxidase over sensitive variety in T_1_, T_2_, T_5_, T_6_, T_7_ and T_10_ whereas, T_8_ and T_9_ were at par for both the sensitive and tolerant varieties. Treatment T_3_ and T_4_ had equal % decrease in ascorbate peroxidase activity in both the varieties. These findings suggest, that PGPR or PGR alone or in combination significantly reduced the reactive oxygen species thus reducing antioxidant enzymes activity in chickpea under water deficit condition. Similar pattern of response was observed during second year (S9 Table).

**Fig 7:**
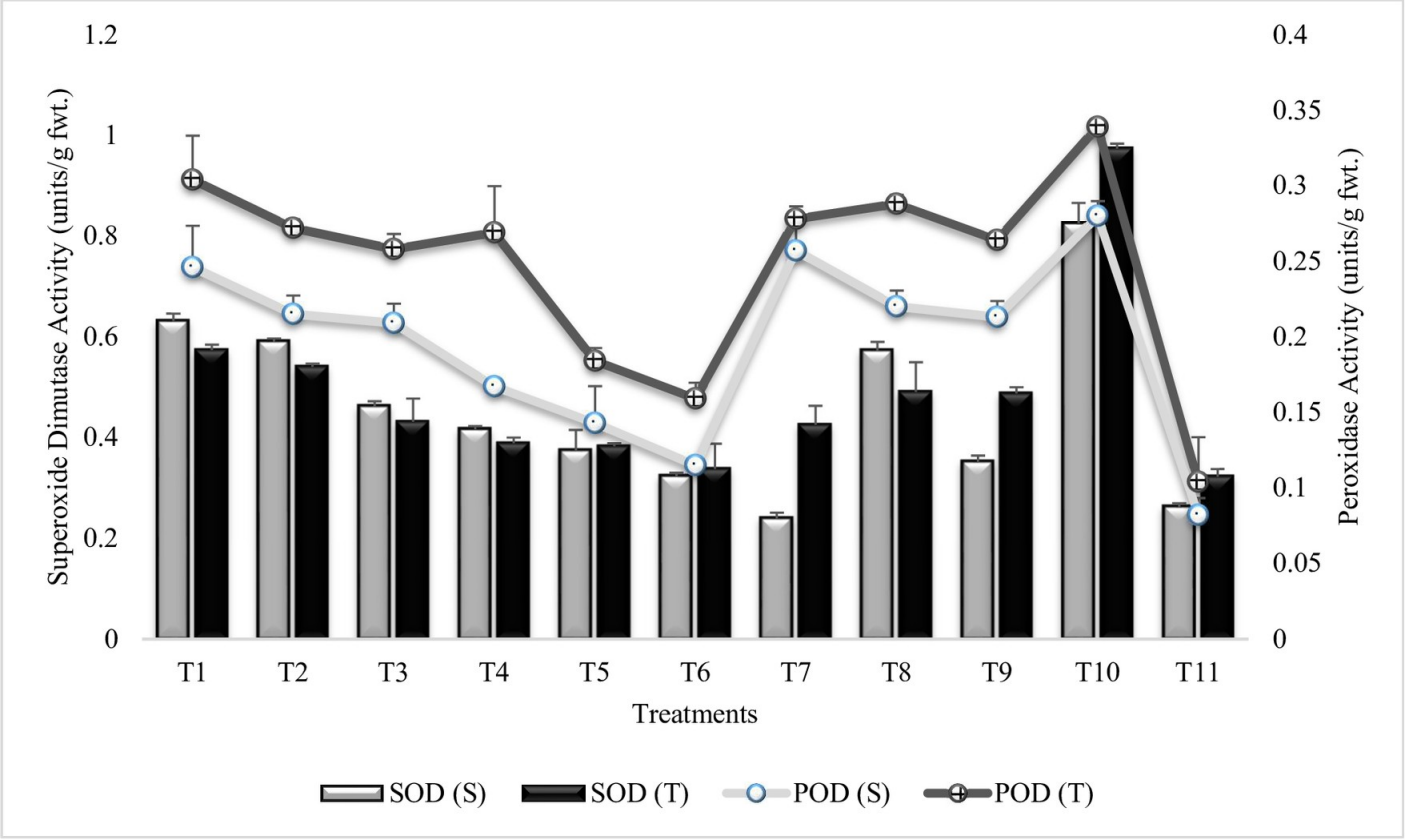
Superoxide dismutase and peroxidase activities of chickpea grown in sandy soil. Data are means of four replicates along with standard error bars (S-Sensitive Variety; T-Tolerant Variety).

In general, the peroxidase activity was decreased in all the inoculated plants as compared to untreated control plants grown in sandy soil (T_10_) however, highly significant decrease (58% and 53%) in peroxidase activity was recorded in T_6_ (Combined treatment of *B*. *subtilis*, *B*. *thuringiensis* and *B*. *megaterium* in combination with SA and Put) followed by T_5_ (Combined treatment of *B*. *subtilis*, *B*. *thuringiensis* and *B*. *megaterium*), for both the sensitive and tolerant varieties over T_10_ (Fig 7). Treatments, T_4_, T_7_ and T_8_ were at par in tolerant variety. The peroxidase activity were decreased with the increase in number of PGPR (T_1_-T_6_). *Bacillus subtilis* (T1) alone was less effective than coinoculation of all three PGPR (T_5_). Plant growth regulators, were more effective in sensitive variety for reducing peroxidase activity when applied alone (T_7_ and T_8_) or in combination (T_9_). Significant reduction in SOD activity was noticed in all the treatments as compared to untreated control plants grown in sandy soil (T_10_). Maximum decrease (72%) was recoded in T_7_ (SA treatment) followed by T_6_ (60%) in sensitive variety whereas, in tolerant variety maximum decrease (65%) was recorded in T_6_ (Fig 7). Treatment T_5_ (Combined treatment of *B*. *subtilis*, *B*. *thuringiensis* and *B*. *megaterium*) and T_6_ (Combined treatment of *B*. *subtilis*, *B*. *thuringiensis* and *B*. *megaterium* in combination with SA and Put) were at par in both the tolerant and sensitive variety, whereas, T_4_ and T_5_ had equal % decrease in SOD activity in tolerant variety. Among the PGR treatments, T_7_ (SA) was more effective and significantly reduced the SOD activity notably, the reduction was even more than irrigated control (T_11_) in the sensitive variety. SA (T_7_) alone or in combination with Put (T_9_) was more responsive for reducing the SOD activity in sensitive variety than tolerant variety. Similar pattern of decrease was followed by all the treatments for POD and SOD activities in the second year (S10 and S11 Tables).

#### Relative water content

**Fig 8:**
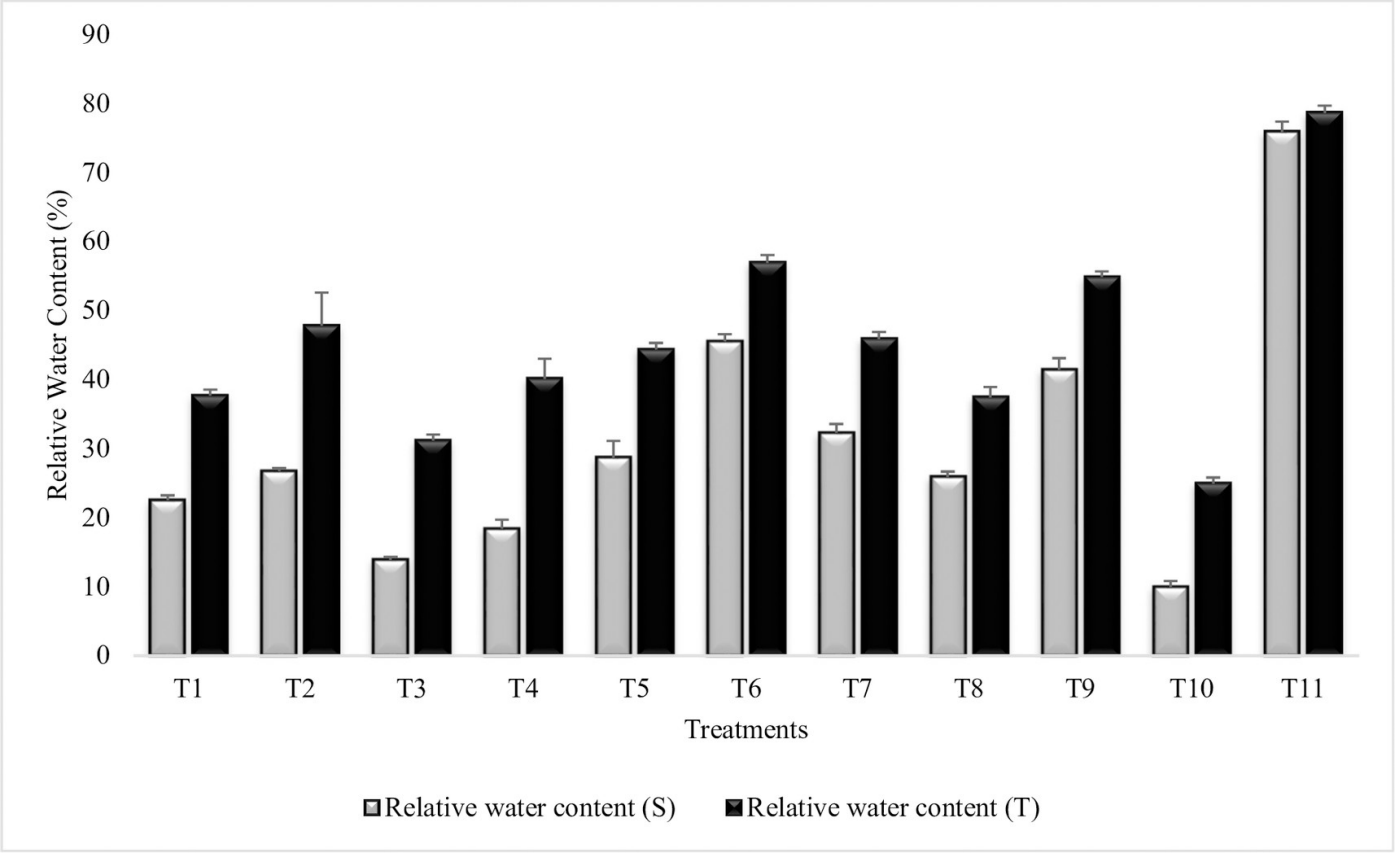
Relative water content of chickpea grown in sandy soil. Data are means of four replicates along with standard error bars (S-Sensitive Variety; T-Tolerant Variety).

All the treatments significantly enhanced Relative Water Content as compared to untreated uninoculated plants grown in sandy soil (T_10_) though the values were lower than irrigated control (T_11_) (Fig 8). Highly significant increase (78% and 56%) in Relative Water Content was recorded in T_6_ (Combined treatment of *B*. *subtilis*, *B*. *thuringiensis* and *B*. *megaterium* in combination with SA and Put) for both the sensitive and tolerant varieties, respectively. In general, tolerant variety had higher values for Relative Water Content in all the treatments over sensitive variety. Treatments T_1_ (*Bacillus subtilis*) and T_8_ (Put treatment) were at par for Relative Water Content in tolerant variety. Treatment T_3_ (Combined treatment of *B*. *thuringiensis* and *B*. *megaterium*) was less effective than T_1_ (*B*. *subtilis* alone) and T_2_ (*B*. *subtilis* in combination with SA and Put). The Relative Water Content of tolerant variety was more (60%) than sensitive variety, in uninoculated untreated plants grown in sandy soil (T_10_). Plant growth regulators, had significantly enhanced the Relative Water Content when applied alone or in combination. Combined treatment of SA and Put (T_9_) was more effective than SA (T_7_) and Put (T_8_) alone. Similar results were reported for Relative Water Content during second year (S12 Table).

#### Shoot fresh weight

**Fig 9:**
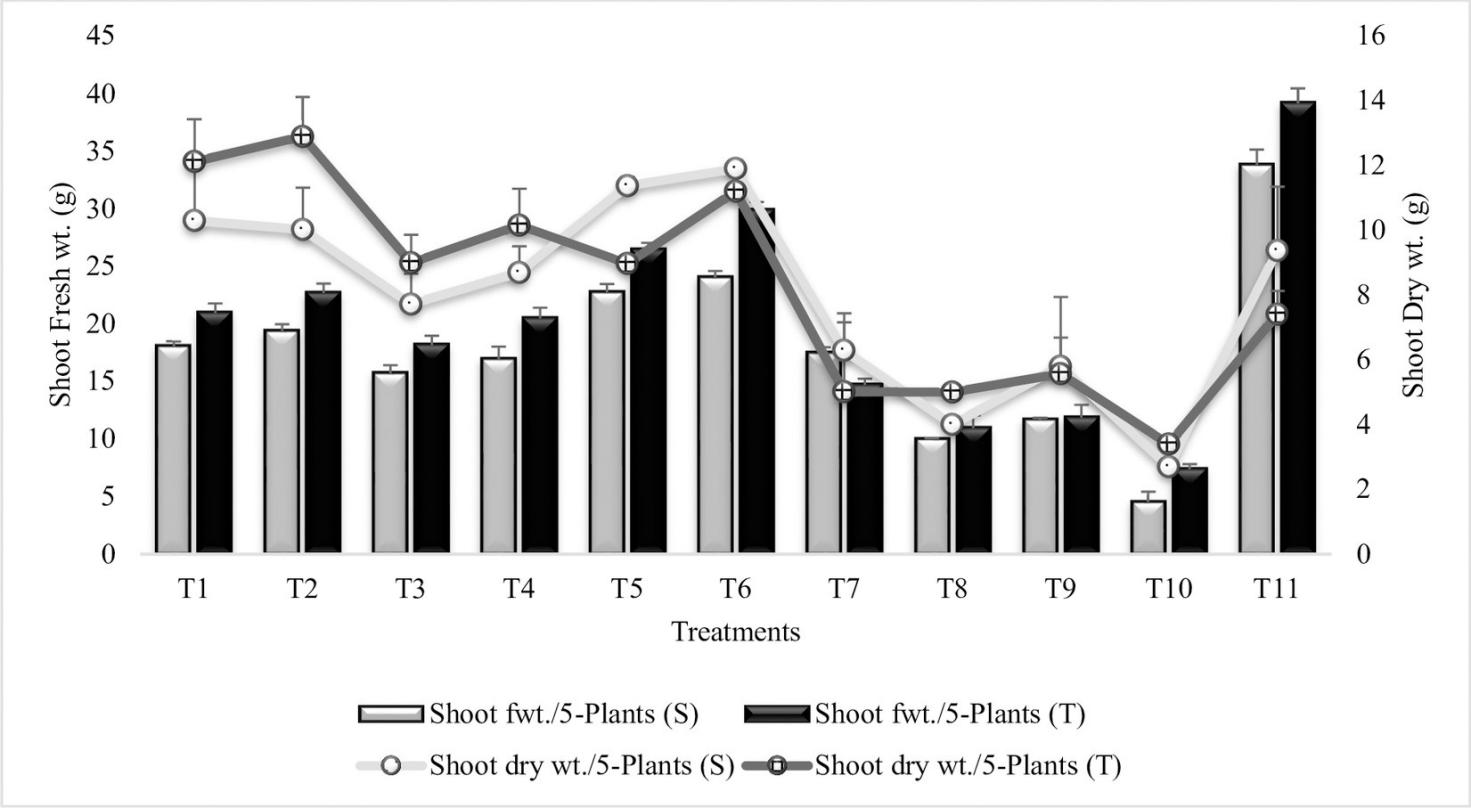
Shoot fresh and dry weights of chickpea grown in sandy soil. Data are means of four replicates along with standard error bars (S-Sensitive Variety; T-Tolerant Variety).

It in inferred from results that shoot fresh weight was significantly increased in all the treatments over untreated plants gown in sandy soil (T_10_) (Fig 9). Maximum increase (81% and 75%) in shoot fresh weight was recorded in T_6_ (Combined treatment of *B*. *subtilis*, *B*. *thuringiensis* and *B*. *megaterium* in combination with SA and Put) followed by T_5_ (Combined treatment of *B*. *subtilis*, *B*. *thuringiensis* and *B*. *megaterium*), in both the sensitive and tolerant varieties. T_1_ (*B*. *subtilis* treatment) and T_4_ (Combined treatment of *B*. *thuringiensis* and *B*. *megaterium* in combination with SA and Put) had equal % increase in shoot fresh weight for both the varieties. The values for shoot fresh weight was higher in tolerant variety over sensitive variety in all the treatments except for T_7_ (SA treatment). Among PGR treatments, SA was more effective for shoot fresh weight than Put (T_8_) or combined treatment of SA and Put (T_9_). Similar results were recorded during the succeeding year 2015–16 (S13 Table).

#### Shoot dry weight

Shoot dry weight was highly significantly increased in all the inoculated treatments over untreated plants grown in sandy soil (T_10_) and over irrigated control (T_11_) (Fig 9). Maximum increase (77%) in shoot dry weight was recorded in T_6_ (Combined treatment of *B*. *subtilis*, *B*. *thuringiensis* and *B*. *megaterium* in combination with SA and Put) for the sensitive variety whereas, in tolerant variety maximum increase (71%) was recorded in T_2_ (P_1_ inoculation in combination with SA and Put). Treatments T_5_, T_6_, T_7_ and T_11_ had higher values in sensitive variety than tolerant variety. *Bacillus subtilis* alone (T_1_) or in combination with PGRs (T_2_) were more effective for shoot dry weight than *B*. *thuringiensis* and *B*. *megaterium* (T3) alone or in combination with SA and Put (T_4_). PGRs were less effective when applied in combination with PGPR. SA (T_7_) was more effective than Put (T_8_) while, combined treatment of SA and Put (T_9_) had similar effect on sensitive and tolerant variety. All the treatments followed similar pattern of response for shoot dry weight during second year except for T_8_ and T_9_ which were reduced (S14 Table).

#### Root fresh weight

**Fig 10:**
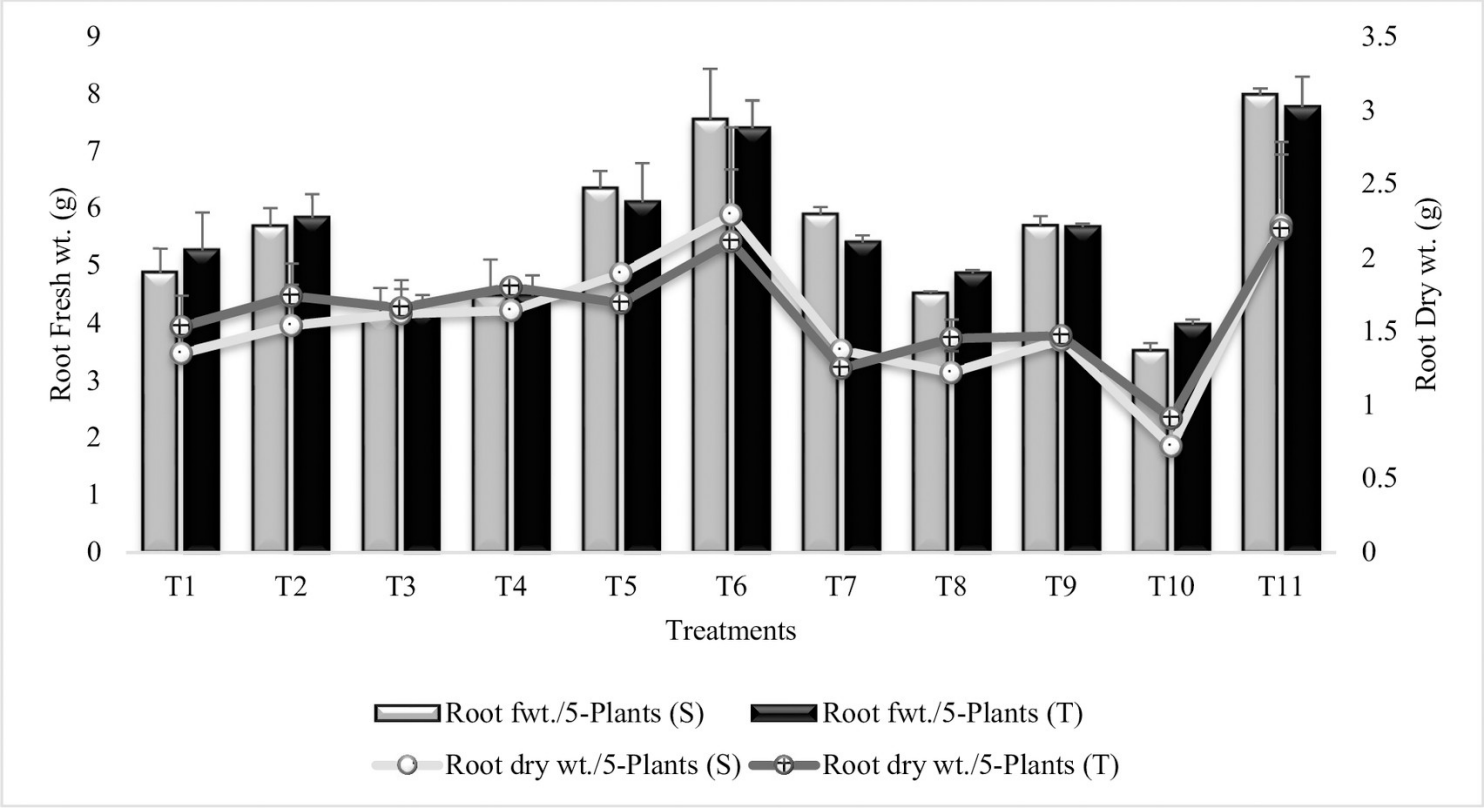
Root fresh and dry weights of chickpea grown in sandy soil. Data are means of four replicates along with standard error bars (S-Sensitive Variety; T-Tolerant Variety).

Root fresh weight was increased in all the treatments over untreated plants grown in sandy soil (T_10_) however, the increase was less than irrigated control (T_11_) (Fig 10). Maximum increase (68% and 56%) in root fresh weight was recorded in T_6_ (Combined treatment of *B*. *subtilis*, *B*. *thuringiensis* and *B*. *megaterium* in combination with SA and Put) in both the tolerant and sensitive varieties. Treatments T_3_ (Combined treatment of *B*. *thuringiensis* and *B*. *megaterium*) and T_4_ (Combined treatment of *B*. *thuringiensis* and *B*. *megaterium* in combination with SA and Put) were less effective than T_1_ (*B*. *subtilis* alone) and T_2_ (*B*. *subtilis* in combination with SA and Put). SA (T_7_) was more effective in sensitive variety whereas, Put (T_8_) was more effective in tolerant variety. Similar results were reported during the second year experiment (S15 Table).

#### Root dry weight

Root dry weight was increased in all the treatments over T_10_ (Fig 10). Maximum increase in root dry weight was recorded in T_6_ (coinoculation of P_1_, P_2_ and P_3_ in combination with SA and Put) in both the sensitive and tolerant varieties. Treatments T_5_, T_6_ and T_7_ had greater values in sensitive variety over tolerant variety. T_3_ (Combined treatment of *B*. *thuringiensis* and *B*. *megaterium*) and T_5_ (Combined treatment of *B*. *subtilis*, *B*. *thuringiensis* and *B*. *megaterium*) had equal % increase in root dry weight for tolerant variety and T_8_ = T_9_ whereas, T_3_ and T_4_ showed equal % increase in sensitive variety. All the PGR treatments showed increase in root dry weight when applied alone (T_7_ and T_8_) or in combination (T_9_). SA was more effective in sensitive variety whereas, tolerant variety was more responsive to Put. Similar, results were recorded during second year (S16 Table).

### Yield and yield contributing characters

#### Number of nodules plant^-1^

**Fig 11:**
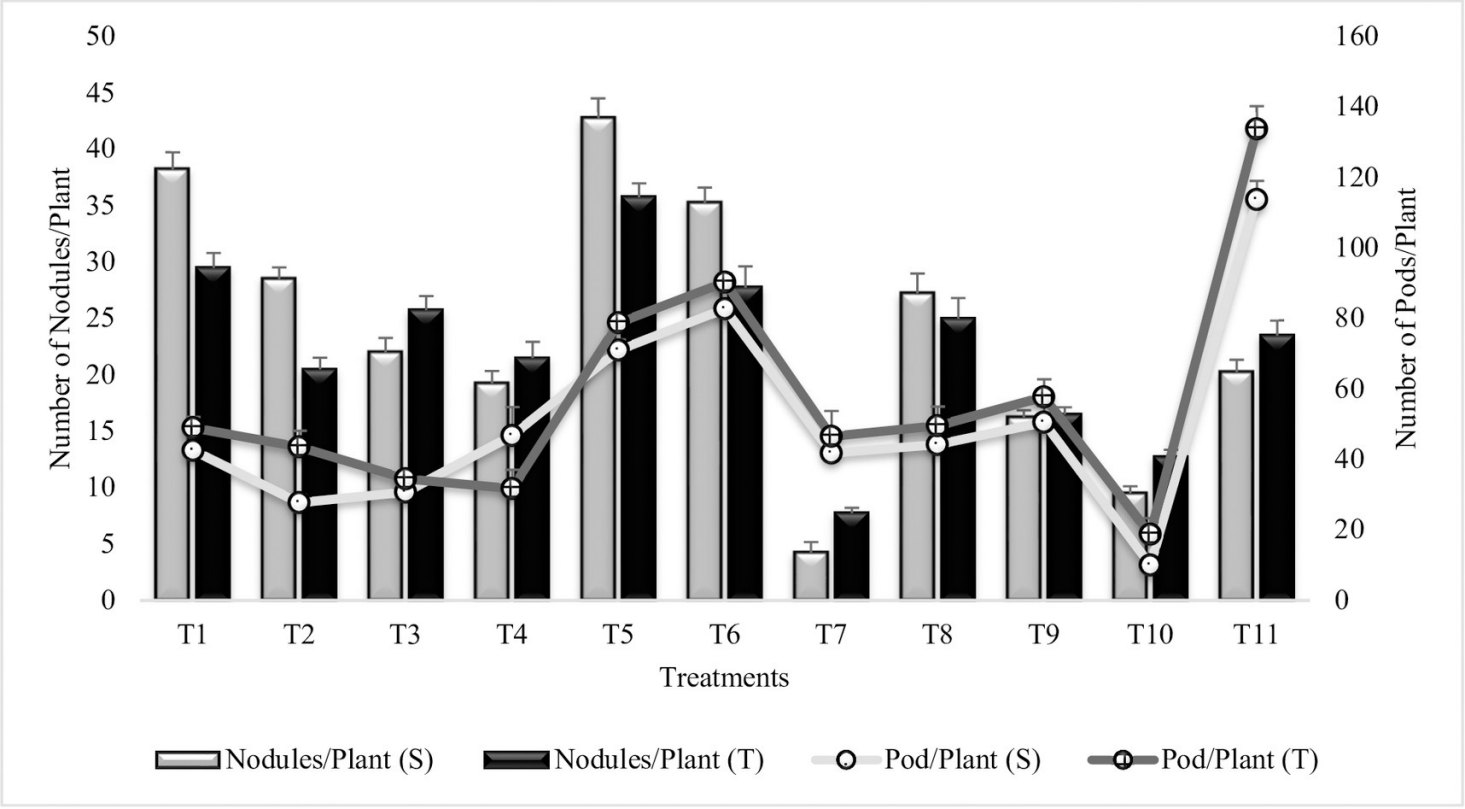
Number of nodules and pods per plant in chickpea grown in sandy soil. Data are means of four replicates along with standard error bars (S-Sensitive Variety; T-Tolerant Variety).

All the treatments significantly enhanced the number of nodules per plant over untreated uninoculated plants grown in sandy soil (T_10_) (Fig 11). Maximum increase (78% and 64%) in number of nodules/plant was recorded in T_5_ (Combined treatment of *B*. *subtilis*, *B*. *thuringiensis* and *B*. *megaterium*) for both the sensitive and tolerant varieties. *Bacillus subtilis* alone (T_1_) instigated highly significant increase (75% and 56%) in number of nodules. In general, the PGPR inoculation was more responsive in sensitive variety than tolerant variety. Combined treatment of *B*. *thuringiensis* and *B*. *megaterium* (T_3_) and putrescine treatment (T_8_) had equal % increase in nodules/plant for tolerant variety whereas, T_5_ in tolerant variety was at par with T_6_ of sensitive variety, similarly T_4_ = T_11_ for sensitive variety. Notably, the values for T_1_, T_3_ and T_4_ in both the varieties were higher than T_2_, T_4_ and T_6_, suggesting the antagonistic effects of SA on number of nodules. SA (T_7_) significantly reduced (55%) the number of nodules but the combined treatment of SA and Put (T_9_) was stimulatory to the number of nodules. Similar results were reported during second year (S17 Table).

#### Number of pods plant^-1^

The number of pods per plant were increased significantly in all the treatments over untreated uninoculated plants grown in sandy soil (T_10_) though the values were lower than irrigated control (T_11_) (Fig 11). Highly significant increase (88% and 79%) was recorded in T_6_ (Combined treatment of *B*. *subtilis*, *B*. *thuringiensis* and *B*. *megaterium* in combination with SA and Put) followed by T_5_ (Combined treatment of *B*. *subtilis*, *B*. *thuringiensis* and *B*. *megaterium*). Tolerant variety, had higher % increase than sensitive variety, except for T_4_ (Combined treatment of *B*. *thuringiensis* and *B*. *megaterium* in combination with SA and Put) which was more effective in sensitive variety. *Bacillus subtilis* alone (T_1_) had equal % increase in pods/plant as compared to T_8_ (Put treatment) in tolerant variety whereas, T_4_ = T_8_ in sensitive variety. PGR, enhanced the number of pods per plant when applied alone or in combination with PGPR except for T_2_. Combined treatment of SA and Put (T_9_) was more effective than SA (T_7_) and Put (T_8_) alone. Similar results were obtained during second year (S18 Table).

#### 100-Pod weight

**Fig 12:**
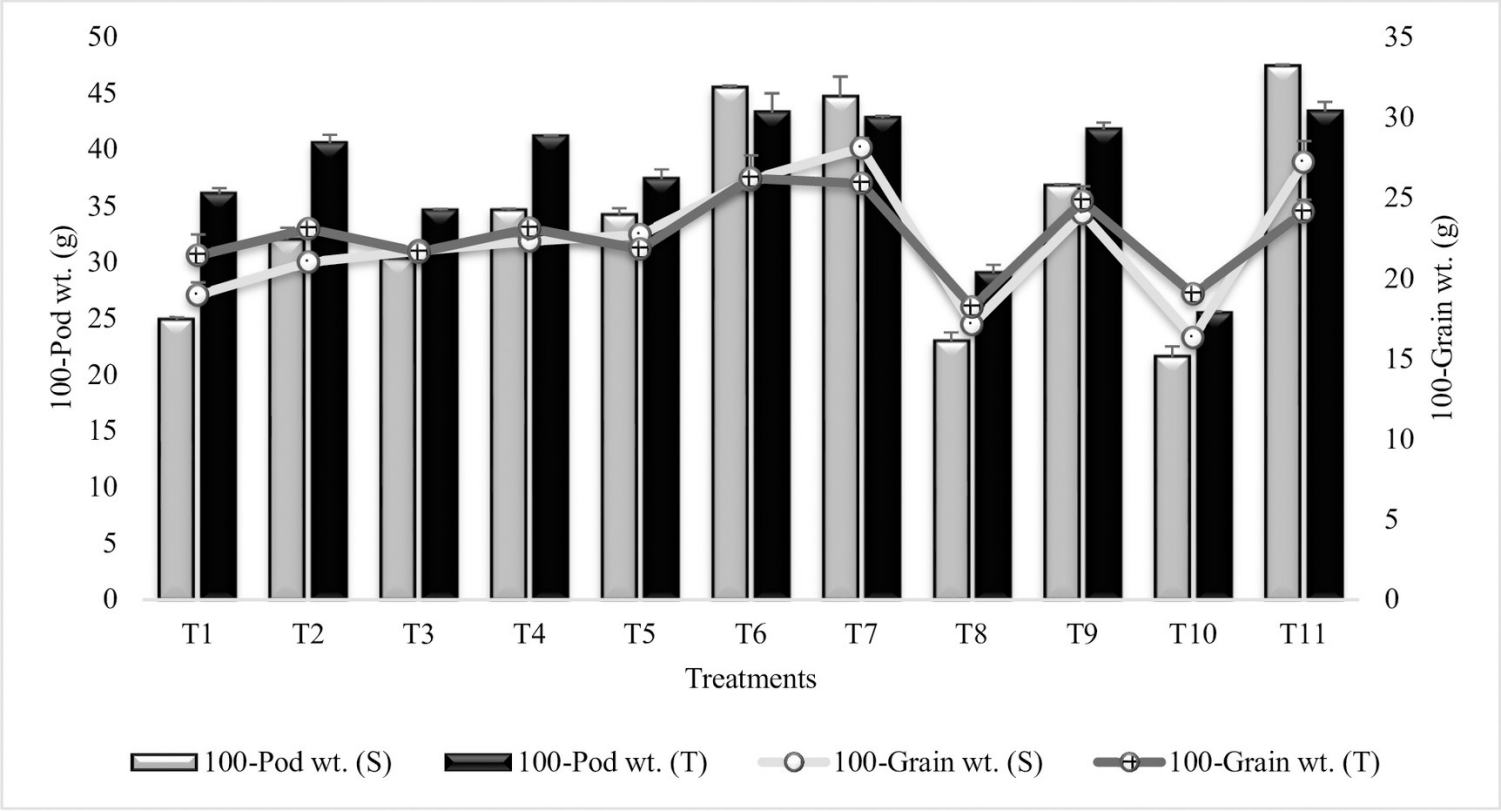
100-pod and grain weight of chickpea grown in sandy soil. Data are means of four replicates along with standard error bars (S-Sensitive Variety; T-Tolerant Variety).

Pod weight was significantly increased in all the treatments as compared to plants grown in sandy soil (T_10_) (Fig 12). Maximum increase (53% and 41%) in 100-pod weight was recorded in T_6_ (Combined treatment of *B*. *subtilis*, *B*. *thuringiensis* and *B*. *megaterium* in combination with SA and Put) for both the sensitive and tolerant varieties, respectively. SA treatment (T_7_) had equal % increase in pod weight as compared to T_6_ and both T_6_ and T_7_ had greater values even more than irrigated control (T_11_) for tolerant variety, while in sensitive variety the % increase was similar to irrigated control. Sensitive variety showed maximum increase over tolerant variety in T_6_, T_7_ and T_11_. T_4_ and T_9_ were at par for 100-pod weight in tolerant variety. SA (T_7_) was more effective among all the PGR treatments and had significantly enhanced (51% and 40%) pod weight, both in sensitive and tolerant varieties as compared to T_10_, suggesting the dominant role of SA on weight of pods. Similar results were reported during second year (S19 Table).

#### 100-Grain weight

Result revealed that maximum increase in 100-grain weight (41%) was due to T_7_ (SA treatment) followed by T_6_ (Combined treatment of *B*. *subtilis*, *B*. *thuringiensis* and *B*. *megaterium* in combination with SA and Put) in sensitive variety whereas, maximum increase (27%) in tolerant variety was recorded in T_6_ followed by T_7_ as compared to untreated plants grown in sandy soil (T_10_) (Fig 12). Notably, T_7_ had greater values than irrigated control (T_11_) in both the varieties whereas, T_6_ had greater values than irrigated control for the tolerant variety. T_5_, T_7_ and T_11_ had greater values for 100-grain weight in sensitive variety than tolerant variety. Combined treatment of *B*. *thuringiensis* and *B*. *megaterium* in combination with SA and Put (T_4_) and Combined treatment of *B*. *subtilis*, *B*. *thuringiensis* and *B*. *megaterium* (T_5_) had equal % increase in sensitive variety whereas, T_6_ and T_7_ had equal % increase in tolerant variety and T_2_ = T_4_. Put (T_8_) alone or in combination with SA (T_9_) was less effective than SA (T_7_) alone, indicating the synergistic effects of SA on 100-grain weight. These results were confirmed from second year data where similar pattern of increase was recorded for all treatments (S20 Table).

#### Plant height

**Fig 13:**
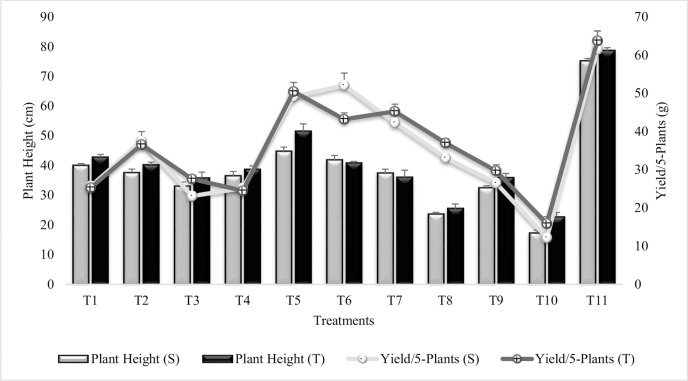
Plant height and yield per 5-plants of chickpea grown in sandy soil. Data are means of four replicates along with standard error bars (S-Sensitive Variety; T-Tolerant Variety).

All the treatments had significantly enhanced the plant height over untreated plants grown in sandy soil (T_10_), though the values were lower than irrigated control (T_11_) (Fig 13). Highly significant increase (61% and 56%) in plant height was recorded in T_5_ (Combined treatment of *B*. *subtilis*, *B*. *thuringiensis* and *B*. *megaterium*) for both the sensitive and tolerant varieties, respectively. *Bacillus subtilis* alone (T_1_) was more effective for increasing plant height than combined treatment of *B*. *thuringiensis* and *B*. *megaterium* (T_3_) and their combination with PGRs (T_4_). Treatments T6 and T7 had greater values in sensitive variety over tolerant variety. T_7_ (SA) alone or in combination with Put (T_9_) significantly enhanced the plant height over T_10_. T_8_ (Put) was less effective when applied alone but showed maximum increase when applied in combination with SA. Plant height followed similar pattern of increase during the succeeding year 2015–16 (S21 Table).

#### Yield per 5-plants

The result revealed that yield/5-plants had significantly enhanced in all the treatments over untreated plants grown in sandy soil (T_10_), (Fig 13). Maximum increase (76%) in Yield per 5-plants was recorded in T_6_ (Combined treatment of *B*. *subtilis*, *B*. *thuringiensis* and *B*. *megaterium* in combination with SA and Put) for sensitive variety followed by T_5_ (coinoculation of P_1_, P_2_ and P_3_) whereas, in tolerant variety, maximum increase (69%) was shown by T_5_ followed by T_6_. T_6_ had greater values for yield/5-plants in sensitive variety than tolerant variety and T_1_ = T_4_ for sensitive variety. Combined treatment of *B*. *thuringiensis* and *B*. *megaterium* (T_3_) was less effective than combined treatment of *B*. *subtilis*, *B*. *thuringiensis* and *B*. *megaterium* (T_5_). SA (T_7_) had significantly enhanced (71% and 64%) yield/5-plants in both sensitive and tolerant varieties as compared to T_10_. Combined treatment of SA and Put (T_9_) was less effective than SA (T_7_) and Put (T_8_) alone, similarly, Put alone (T_8_) was less effective than SA (T_7_). Similar results were recorded during succeeding year 2015–16 (S22 Table).

#### Total biomass

**Fig 14:**
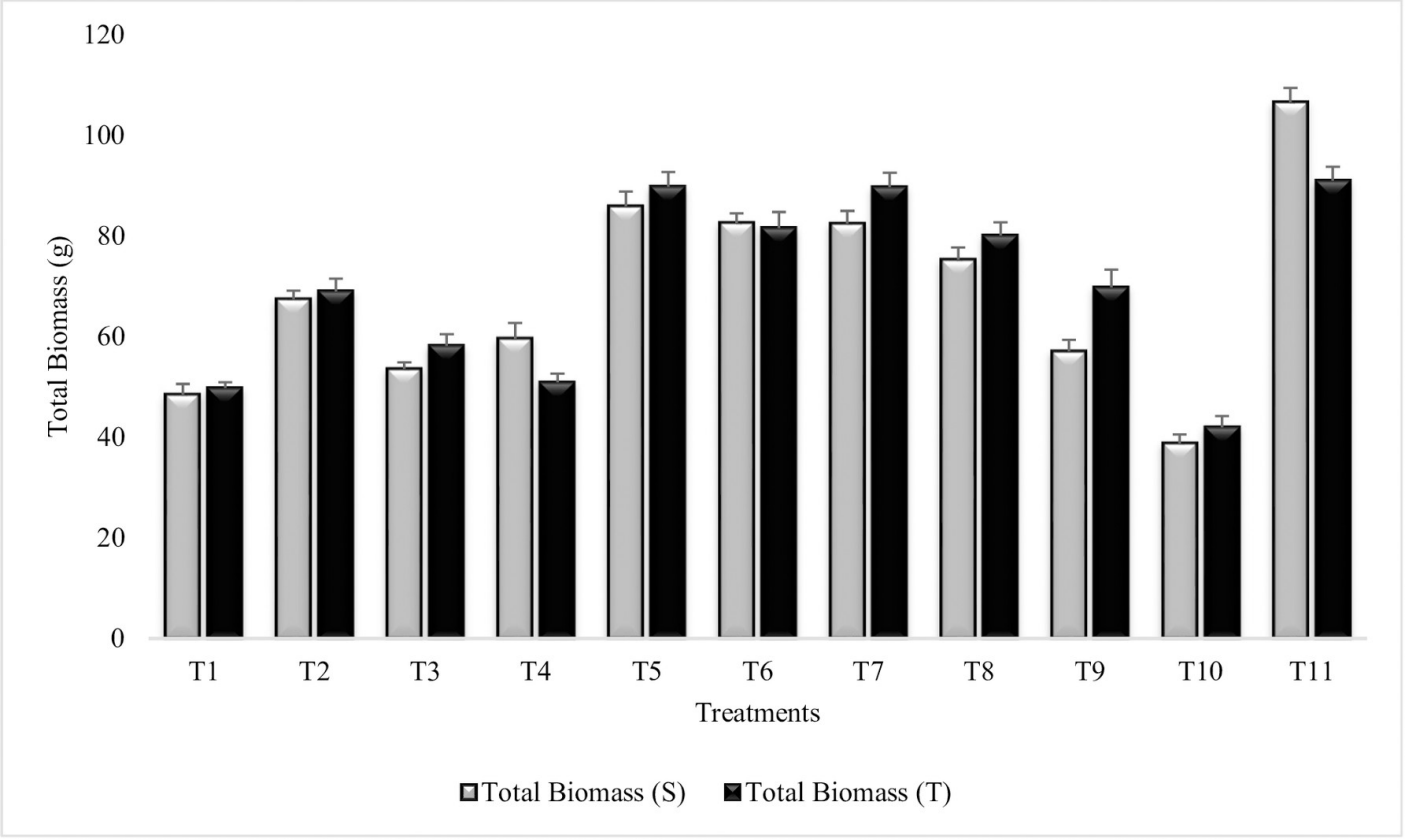
Total biomass of chickpea grown in sandy soil. Data are means of four replicates along with standard error bars (S-Sensitive Variety; T-Tolerant Variety).

Results revealed significant increase in total biomass in treated plants over untreated control plants grown in sandy soil (T_10_). Maximum increase (54% and 53%) in total biomass was recorded in T_5_ (Combined treatment of *B*. *subtilis*, *B*. *thuringiensis* and *B*. *megaterium*) followed by T_7_ (SA treatment) for both the sensitive and tolerant varieties, respectively (Fig 14). Notably, T_1_ (*B*. *subtilis* alone), T_2_ (*B*. *subtilis* in combination with SA and Put) and T_6_ had equal % increase in both the varieties. Combined treatment of *B*. *thuringiensis* and *B*. *megaterium* in combination with SA and Put (T_4_) and irrigated C (T_11_) had greater values for total biomass in sensitive variety over tolerant variety. SA (T_7_) was more effective among all the PGR treatments and had significantly enhanced (52% and 53%) the total biomass. Combined treatment of SA and Put (T_9_) was less effective than SA (T_7_) and Put (T_8_) alone. Similar results were reported during succeeding year 2015–16 (S23 Table).

#### Harvest index

**Fig 15:**
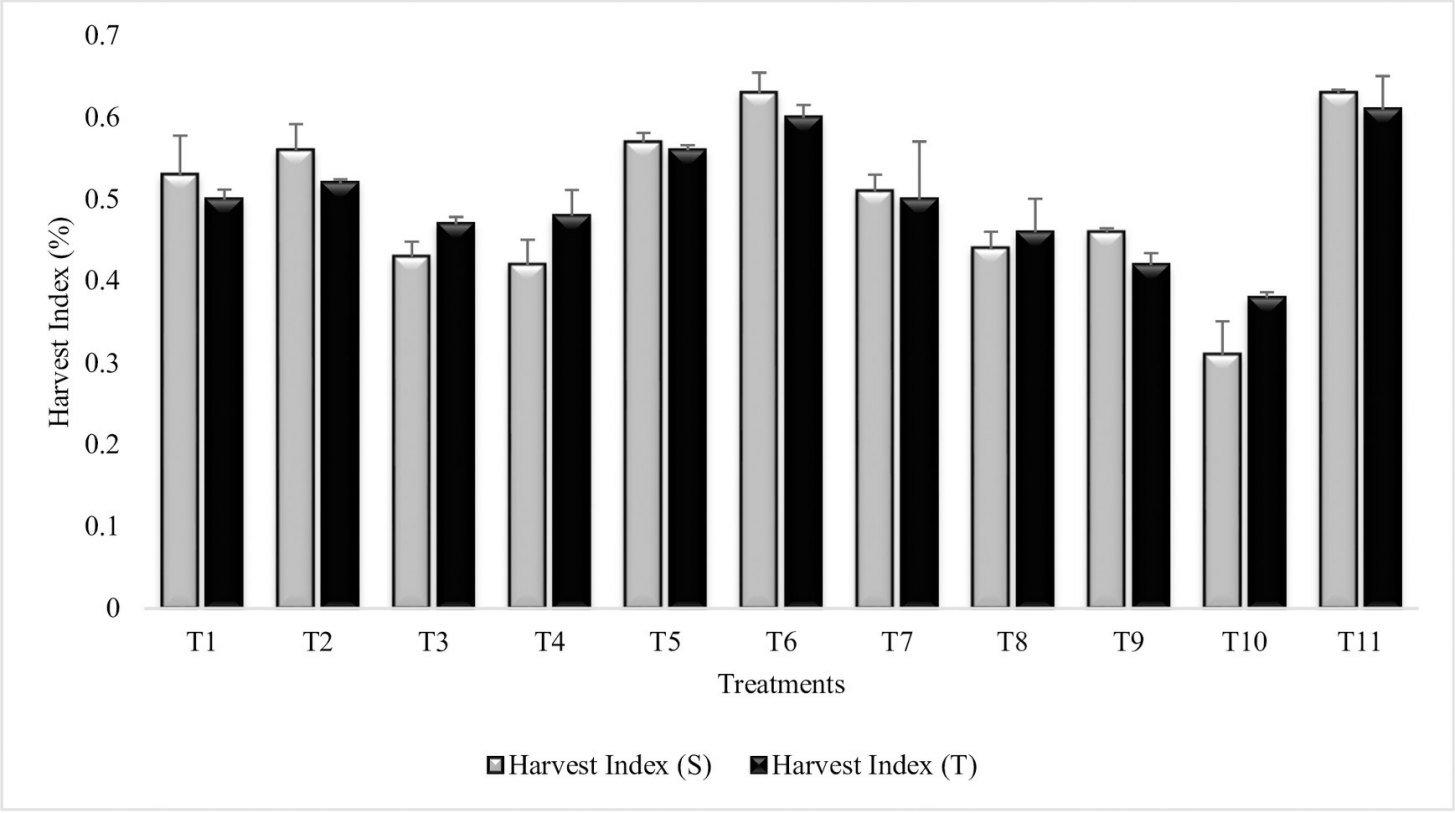
Harvest index of chickpea grown in sandy soil. Data are means of four replicates along with standard error bars (S-Sensitive Variety; T-Tolerant Variety).

All the treatments had significantly enhanced harvest index over untreated plants grown in sandy soil (T_10_). Maximum increase in harvest index was recorded in T_6_ (Combined treatment of *B*. *subtilis*, *B*. *thuringiensis* and *B*. *megaterium* in combination with SA and Put) for both the sensitive and tolerant varieties (Fig 15). T_6_ was at par with irrigated control (T_11_). Sensitive variety showed maximum increase in T_1_, T_2_, T_6_, T_9_ and T_11_ over tolerant variety. Combined treatment of *B*. *thuringiensis* and *B*. *megaterium* (T_3_) and their combination with PGRs (T_4_) enhanced the harvest index for both the sensitive and tolerant varieties. SA alone (T_7_) was more effective than Put (T_8_) or combined treatment of SA and Put (T_9_). It was also inferred from results that combined treatment of SA and Put (T_9_) had antagonistic effects on tolerant variety. Similar results were reported during second year experiment (S24 Table).

## Discussion

Moisture stress is one of the major restraint for agricultural crops that not only affect plant physiology but productivity [56, 57]. Approximately, 40% of the agricultural lands lies at arid and semi-arid regions of the world. Moisture stress effects the morphological and physiological characteristics, and also have negative impacts on fresh weights, relative water content and decrease nutrient diffusion. Plant growth promoting rhizobacteria (PGPR) and plant growth regulators (PGR) could play a significant role in alleviation of moisture stress in plants.

Chlorophyll content was increased in all the treatments but maximum increase was recorded in T_6_ and T_5_ for both the field and pot experiments. This increase may be due to the synergistic effects of PGPR consortia and PGR, salicylic acid (SA) + Putrescine (Put) which was at par to irrigated control. Thereby, suggesting that combined treatment of PGR and PGPR can effectively ameliorate the adverse effects of moisture stress. It had been reported previously that PGPR induce chlorophyll content in many plants grown under abiotic stress condition [58]. Kumar *et al*. [59] reported decrease in chlorophyll content in the leaves of chickpea due to drought stress however; inoculation with PGPR amended the adverse effects of drought on chlorophyll content. It had been previously reported that SA significantly enhanced the chlorophyll content in many crop plants [60, 61]. Previous studies suggest the significantly positive effects of Put on leaf chlorophyll content and was helpful in preventing the degradation of chlorophyll due to abiotic stresses [62, 63].

It is inferred from the results that proline production was decreased in the PGPR consortia inoculated plants which possibly demonstrate the mitigation ability of the osmotic stress and maintenance of bioenergetics of cell in moisture stress condition. Further decrease in proline content was obvious in the combined treatment (T_6_) of PGR and PGPR keeping it at par to the irrigated control. This indicating that PGR + PGPR effectively protect the Put from secondary stresses (osmotic stress) created by the moisture stress induced by sandy soil. Similar, results had also been reported by Jha *et al*. [64], that proline accumulation increased with salinity but decreased in plants inoculated with *Pseudomonas pseudoalcaligenes* and *B*. *pumilus* alone or in combination. The role of PGR on proline had also been reported earlier [65, 66]. Su and Bai [67] studied the accumulation of proline in soyabean leaves grown under stress condition and found a negative correlation between accumulation of proline and endogenous Put content; enhanced accumulation of Put lead to decrease in proline content.

The coinoculation of 3-PGPR namely, *Bacillus subtilis*, *Bacillus thuringiensis* and *Bacillus megaterium* had significantly enhanced the leaf protein content in drought tolerant and sensitive varieties. New proteins possibly appear to be synthesized in stressed plants grown in sandy soil (T_10_) and the tolerant variety had greater potential for this. However, sensitive variety was more effective under irrigated condition. Plants normally synthesize heat shock proteins, antioxidant enzymes and several plant hormones to cope with environmental stresses. Noteworthy, the PGPR (T_5_) significantly enhanced the protein content. Dashti *et al*. [68] indicated that co-inoculation of soybean with *B*. *japonicum* and *Serratia species* increased grain yield, protein yield, and total plant protein content. Afzal and Bano [69] reported PGPR induced increase in leaf protein content of wheat. Similar results were reported by Islam *et al*. [70] and Pérez-Montaño *et al*. [71] in cereal and leguminous plants. The additive effect of SA on leaf protein content had been reported previously by Neelam *et al*. [72]. Çanakci and Dursun, [73] reported increase in protein content in leaves of chickpea treated with SA. Put induced increase in protein content had also been reported previously by many authors [74, 75].

In present study, a significant change in leaf sugar content was obvious in inoculated plants. The maximum sugar accumulation in the leaves of T_5_ (coinoculation of P_1_, P_2_ and P_3_), demonstrated the better mechanism for osmo-adjustment. It was noted that the sensitive variety was more responsive to sugar accumulation. Plant growth regulators (SA and Put) had no or little affect when applied to inoculated plants but had significantly enhanced the sugar content when applied alone. Environmental stresses had significantly decreased the leaf sugar content thus causes, physiological and biochemical alterations as sugar preserve the structure of macromolecules and membranes during extreme dehydration [76]. It had been reported that PGPR-accumulated soluble sugars may lead to drought tolerance in plants [77]. Beneficial effects of PGPR on root sugar content had also been reported previously [78, 79]. It is supposed that SA treatment disturbs the enzymatic system of polysaccharide hydrolysis and thus lead to increase sugar level which may lead to osmotic adjustment under stress condition [80]. The role of Put in accumulation of sugar in plant leaves under stress condition had also been reported previously by many workers [81, 82].

The suppressive effects of PGPR and more so by PGPR + PGR is noteworthy for reducing the lipid peroxidation as measured by the malondialdehyde (CMDA) content of the leaves. It can be inferred from the result that similar characteristic exist for PGR (SA + Put) both in terms of proline content and lipid peroxidation content of leaves. Lipid peroxidation act as biomarker for tissues and membrane damage under stress condition. Increase in lipid peroxidation is considered as indication for increase in oxidative damage. Singh and Jha *et al*. [83] recorded an increase in lipid peroxidation in wheat with the increase in salt concentration however, inoculation with PGPR significantly reduced the lipid peroxidation in salt treated plants. This decrease in lipid peroxidation with PGPR inoculation may be attributed to the fact that PGPR inoculation lower cell injuries caused by abiotic stresses and increase tolerance to environmental stresses. Coinoculation of *Pseudomonas pseudoalcaligenes* and *Bacillus pumilus* had significant adverse effects on lipid peroxidation in paddy grown under salt stress condition [64]. Put reduce oxidative damages by reducing lipid peroxidation had been reported earlier by Tang *et al*. [84].

Antioxidant enzymes play a critical role in detoxifying the harmful effects of reactive oxygen species, produced in response to environmental stresses. However, PGPR inoculation reduced the antioxidant enzyme activities in all inoculated treatments and the addition of PGR further reduced the antioxidant enzyme activities. This decrease in antioxidant enzyme activities with PGPR inoculation may be attributed to the fact that PGPR ameliorated the harmful effects of moisture stress hence, reducing the production of reactive oxygen species. It is inferred from results that inoculation of chickpea with PGPR could provide drought tolerance ability by reducing the harmful effects of reactive oxygen species. These results were in agreement with those reported by Jha and Subramanian [64] that the inoculation with PGPR strains reduced the lipid peroxidation and superoxide dismutase (SOD) activity in sensitive cultivars of *Oryza sativa* and endorsed resistance to salt stress. PGPR induced decrease in antioxidant enzyme activity had been reported previously in many plants including canola, cucumis, wheat and barley [85, 86]. It has been observed that exogenous application of SA and Put can regulate the activities of intracellular antioxidant enzymes such as SOD, ascorbate peroxidase (APOX) and increase plant tolerance to environmental stresses [87, 88].

Plant phenolics are secondary metabolites that play an imperative role in growth and reproduction and help plant to withstand under severe stress condition. It is inferred from result that phenolic content had been increased significantly in the leaves of inoculated plants. Coinoculation of all 3-PGPR and 2-PGR had significantly augmented phenolic content of both the sensitive and tolerant varieties and hence increase the plant tolerance to moisture stress. These results were in agreement with those reported by Bahadur *et al*. [89], who noted an increase in phenolic content in the leaves of pea plants inoculated with PGPR strains and with the results of Chakraborty *et al*. [90] who also reported PGPR induced increase in phenolic content. Combined treatment of SA and Put had significantly enhanced the phenolic content and was more responsive in sensitive variety, indicating the role of PGR in drought tolerance. These results were in agreement with the findings of War *et al*. [91], who reported increase in phenolic contents of chickpea sprayed with SA. Similar results had also been reported previously by many workers [72, 92]. Put increase, phenolic content of Gladiolus, Chamomilla and wheat had been documented earlier [93, 94].

Relative Water Content reflects a measure of plant water status, which in turn is used as an index for dehydration tolerance. Decrease in Relative Water Content in untreated uninoculated plants grown in sandy soil was obvious during the present research however, PGPR inoculation overcome the water deficit induced reduction in Relative Water Content. These results correspond to that of Casanovas *et al*. [95] in maize seedlings, inoculated with *A*. *brasilense* that lead to improved relative and absolute water contents compared to uninoculated plants grown under moisture stress. Inoculation with *Pseudomonas putida* improved plant biomass, Relative Water Content and leaf water potential in maize plants exposed to moisture stress [96]. PGPR induce increase in Relative Water Content had been studied by many other workers [97]. This increase in relative water content by PGPR inoculation was attributed to the PGPR induced product of plant hormones such as IAA by the bacteria that improved root growth and formation of lateral roots their by increasing uptake of water and nutrients under moisture stress [98].

The coinoculation of 3-PGPR (P_1_, P_2_ and P_3_) alone or in combination with PGR (SA and Put) had significantly enhanced shoot fresh and dry weights and these treatments were more responsive in sensitive variety than tolerant variety. These results were confirmed by Ahemad and Kibret [99] who reported enhanced shoot fresh and dry weights in *Brassica* plants inoculated with PGPR. Similar results were reported by Islam *et al*. [100] in cucumber and by Huang *et al*. [101] in corn, pepper and tomato. SA induced increase in shoot fresh and dry weights had been well documented in many plants including, Ocimum, lemongrass, sunflower, strawberry, wheat and maize [102]. Results of an experiment conducted by Ahmed *et al*. [103] indicated that Put application significantly enhanced shoot fresh and dry weights of cotton grown under abiotic stress condition.

In the present study root area was highly significantly enhanced in plants inoculated with consortium of 3 PGPR. The observed increase in root area in SA and Put treated plants may be attributed to SA and Put modulation of stimulating phytohormones IAA and cytokinin. Noteworthy, the sensitive variety was more responsive to both PGPR and PGR for root parameters. PGPR stimulated root growth because of their ability to produce IAA and IAA had long been known for its stimulatory effects on root parameters [104]. Erturk *et al*. [105] reported 47% increase in rooting ratios when plants were inoculated with PGPR strains. Gamalero *et al*. [106] reported the impact of two *fluorescent pseudomonads* and an arbuscular mycorrhizal fungus on the growth and root architecture of tomato plant and observed enhanced root growth in inoculated treatments. This increase in root parameters play an important role in developing tolerance to abiotic stresses especially to moisture stress because enhanced root length promote plant growth during stress condition [107]. Zhu *et al*. [108] pointed out the synergistic effects of Put on the shoot and root growth of chickpea and soyabean under stress condition.

Coinoculation of 3-PGPR significantly enhanced number of nodules per plant. However, P_1_ inoculation alone was much effective in increasing number of nodules per plant. This increase in number of nodules was due to the fact, that PGPR has positive effects on the symbiotic performance of rhizobia that cause more nodulation. Nodulation is of great importance for N-fixation, play an important role in plant growth and productivity and enhance tolerance to adverse environmental factors [109]. Co-inoculation of plant growth promoting rhizobacteria (PGPR) with *Bradyrhizobium* had been shown to increase legume nodulation and nitrogen fixation in alfalfa, soyabean and common bean [110]. Similarly, legume growth and yield have been shown to increase in inoculated plants. Bai *et al*. [111] reported that the co-inoculation of *Bacillus* strains in soybean plants with *Bradyrhizobium japonicum* showed the highest increase in nodule number, nodule weight, shoot and root weights, total biomass, total nitrogen, and grain yield. Put induced increase in nodules had been reported previously [112].

It is inferred from results that the number and weight of pods have been increased in inoculated plants. Combination of 3-PGPR and 2-PGR was more effective and significantly enhanced the pod number and weight. It has been reported that *Pseudomonas fluorescens* and *Azospirillum lipoferum* significantly increased pods per plant, weight of pod and total dry matter in *Phaseolus vulgaris* [113]. Dey *et al*. [114] reported the significant increase in number of pods and pod weight in peanut plants inoculated with seven different *pseudomonas* species. Combined inoculation of *Escherichia coli*, *Pseudomonas fluorescens* and *Burkholderia sp*. significantly enhanced the pod bearing branches, pod/plant and weight of pod in chickpea plants [115]. SA alone had significantly enhanced pod and grain weight and the pods matured late in SA treated plants. These results are in support by those of El-Hak *et al*. [116] who found that foliar application of SA significantly increased pod and grain weight in pea plant.

The combined treatments of SA and Put was more effective for increase in yield and yield related components (yield/5-plants, total biomass and harvest index) of chickpea grown in sandy soil. Several studies have currently exposed that inoculation with PGPR, increased leaf area, growth and yield in many plants including legumes [117, 118]. PGPR can enhance plant growth and yield either by increasing leaf area, nitrogen uptake, phytohormone production, minerals solubilisation or by chelation of iron [119]. Beside this, PGPR induced increase in yield had been studied in many other plants including sweet potato, apple, tomato, maize and peanut [120, 121].

## Conclusion

The PGPR and/or PGR showed increase in all growth parameters but the magnitude of increase was higher in their combined treatment. The combined effect of PGR with PGPR was to decrease PGPR induced decrease in antioxidant enzymes, proline production and lipid per oxidation as measured by MDA content of leaves. Moisture stress significantly reduced the physiological parameters but the inoculation of PGPR and PGR treatment effectively ameliorated the adverse effects of moisture stress. Significant increase in the content of secondary metabolites was noted in the leaves of all treated plants but the content of antioxidant enzymes were decreased in reaction to PGPR and PGR treatments that lead to systematic acquired resistance. PGPR and/or PGR treatment was also helpful for increase in plant height, grain weight number of nodule and pods that lead to increase in plant yield. It is therefore, concluded that the integrative use of active PGPR strains (biotic elicitors) and PGRs seems to be a promising method and eco-friendly strategy that will help to reduce the harmful effects of moisture stress on crop plants cultivated in arid regions all over the world.

## Supporting information

S1 Table Effect of PGPR inoculation and PGR treatment alone or in combination on leaf chlorophyll content (mg/g) of chickpea grown in sandy soil. (DOCX)

S2 Table Effect of PGPR inoculation and PGR treatment alone or in combination on leaf proline content (ug/g) of chickpea grown in sandy soil. (DOCX)

S3 Table Effect of PGPR inoculation and PGR treatment alone or in combination on leaf protein content (ug/g) of chickpea grown in sandy soil. (DOCX)

S4 Table Effect of PGPR inoculation and PGR treatment alone or in combination on lipid peroxidation (nmol/g fwt.) of chickpea grown in sandy soil. (DOCX)

S5 Table Effect of PGPR inoculation and PGR treatment alone or in combination on leaf sugar content (mg/g) of chickpea grown in sandy soil. (DOCX)

S6 Table Effect of PGPR inoculation and PGR treatment alone or in combination on root sugar content (mg/g) of chickpea grown in sandy soil. (DOCX)

S7 Table Effect of PGPR inoculation and PGR treatment alone or in combination on leaf phenolic content (mM GA eq/g FW) of chickpea grown in sandy soil. (DOCX)

S8 Table Effect of PGPR inoculation and PGR treatment alone or in combination on catalase activity (units/g fwt.) in the leaves of chickpea grown in sandy soil. (DOCX)

S9 Table Effect of PGPR inoculation and PGR treatment alone or in combination on ascorbate peroxidase (APOX) activity (units/g fwt.) in the leaves of chickpea grown in sandy soil. (DOCX)

S10 Table Effect of PGPR inoculation and PGR treatment alone or in combination on peroxidase activity (units/g fwt.) in the leaves of chickpea grown in sandy soil. (DOCX)

S11 Table Effect of PGPR inoculation and PGR treatment alone or in combination on superoxide dismutase activity (units/g fwt.) in the leaves of chickpea grown in sandy soil. (DOCX)

S12 Table Effect of PGPR inoculation and PGR treatment alone or in combination on relative water content (%) of chickpea grown in sandy soil. (DOCX)

S13 Table Effect of PGPR inoculation and PGR treatment alone or in combination on shoot fresh weight (g) of chickpea grown in sandy soil. (DOCX)

S14 Table Effect of PGPR inoculation and PGR treatment alone or in combination on shoot dry weight (g) of chickpea grown in sandy soil. (DOCX)

S15 Table Effect of PGPR inoculation and PGR treatment alone or in combination on root fresh weight (g) of grown in sandy soil. (DOCX)

S16 Table Effect of PGPR inoculation and PGR treatment alone or in combination on root dry weight (g) of chickpea grown in sandy soil. (DOCX)

S17 Table Effect of PGPR inoculation and PGR treatment alone or in combination on number of nodules per plant of chickpea grown in sandy soil. (DOCX)

S18 Table Effect of PGPR inoculation and PGR treatment alone or in combination on number of pods per plant of chickpea grown in sandy soil. (DOCX)

S19 Table Effect of PGPR inoculation and PGR treatment alone or in combination on 100-pod weight (g) of chickpea grown in sandy soil. (DOCX)

S20 Table Effect of PGPR inoculation and PGR treatment alone or in combination on 100-grain weight (g) of chickpea grown in sandy soil. (DOCX)

S21 Table Effect of PGPR inoculation and PGRs treatment alone or in combination on plant height (cm) of chickpea grown in sandy soil. (DOCX)

S22 Table Effect of PGPR inoculation and PGR treatment alone or in combination on yield per 5-plants (g) of chickpea grown in sandy soil. (DOCX)

S23 Table Effect of PGPR inoculation and PGR treatment alone or in combination on total biomass (g) of chickpea grown in sandy soil. (DOCX)

S24 Table Effect of PGPR inoculation and PGR treatment alone or in combination on harvest index (%) of chickpea grown in sandy soil. (DOCX)
