# Correction: Impacts of plant growth promoters and plant growth regulators on rainfed agriculture

**DOI:** 10.1371/journal.pone.0232926

**Published:** 2020-05-04

**Authors:** The PLOS ONE Staff

The second and third authors’ names are spelled incorrectly. The correct names are: Asghari Bano and M.D. Ali Babar. The correct citation is: Khan N, Bano A, Babar MDA (2020) Impacts of plant growth promoters and plant growth regulators on rainfed agriculture. PLoS ONE 15(4): e0231426. https://doi.org/10.1371/journal.pone.0231426.

The word “Biosciences” is misspelled in the second affiliation. The correct affiliation is: Department of Biosciences, University of Wah, Wah Cantt, Pakistan.

The publisher apologizes for the errors.
