# Supplementary material for: Impacts of plant growth promoters and plant growth regulators on rainfed agriculture
Source: PLoS One. 2020 Apr 9;15(4):e0231426. doi: 10.1371/journal.pone.0231426 (PMC7145150; doi:10.1371/journal.pone.0231426)
Supplement: S1 Table — (DOCX) [file pone.0231426.s001.docx]

**S1 Table. Effect of PGPR inoculation and PGR treatment alone or in combination on leaf chlorophyll content (mg/g) of chickpea grown in sandy soil.**

| **Treatments** | **2014-15 (S)** | **2015-16 (S)** | **Mean** | **2014-15**  **(T)** | **2015-16 (T)** | **Mean** |
| --- | --- | --- | --- | --- | --- | --- |
| T1 | 40.2 e | 41.7 c | 40.9 | 36.6 df | 39.7 e | 38.1 |
| T2 | 42.2 de | 43.5 c | 42.8 | 40.9 cd | 42.6 de | 41.7 |
| T3 | 44.1 cd | 46 bc | 42.85 | 43.2 bc | 44.6 cd | 43.9 |
| T4 | 48.4 b | 49.4 b | 48.9 | 44.4 bc | 46.9 bcd | 45.6 |
| T5 | 47.1 bc | 50.2 b | 48.6 | 44.9 bc | 48.3 abc | 46.6 |
| T6 | 55.5 a | 56.2 a | 55.8 | 50.1 a | 52.6 a | 51.3 |
| T7 | 39.2 e | 41.9 c | 40.5 | 37.7 d | 38.4 ef | 38 |
| T8 | 32.8 f | 35.8 d | 34.3 | 32 ef | 35.1 f | 33.5 |
| T9 | 44.5 cd | 45.9 bc | 45.2 | 42.6 c | 45.2 cd | 43.9 |
| T10 | 22.3 g | 25.9 e | 24.1 | 27.4 f | 28.8 g | 28.1 |
| T11 | 57.5 a | 58.5 a | 58 | 47.5 ab | 50.8 ab | 49.1 |

Values followed by different letters in a column were significantly different (P<0.005). Data are average of four replicates (S- Sensitive Variety, T-Tolerant Variety).
