# Supplementary material for: Impacts of plant growth promoters and plant growth regulators on rainfed agriculture
Source: PLoS One. 2020 Apr 9;15(4):e0231426. doi: 10.1371/journal.pone.0231426 (PMC7145150; doi:10.1371/journal.pone.0231426)
Supplement: S2 Table — (DOCX) [file pone.0231426.s002.docx]

**S2 Table. Effect of PGPR inoculation and PGR treatment alone or in combination on leaf proline content (ug/g) of chickpea grown in sandy soil.**

| **Treatments** | **2014-15 (S)** | **2015-16 (S)** | **Mean** | **2014-15 (T)** | **2015-16 (T)** | **Mean** |
| --- | --- | --- | --- | --- | --- | --- |
| T1 | 0.202 cde | 0.206 e | 0.2 | 0.242 bcd | 0.236 d | 0.23 |
| T2 | 0.17 e | 0.181 f | 0.17 | 0.233 bcd | 0.223 e | 0.23 |
| T3 | 0.272 ab | 0.287 b | 0.27 | 0.274 b | 0.293 b | 0.28 |
| T4 | 0.212 cde | 0.235 c | 0.22 | 0.234 bcd | 0.26 c | 0.24 |
| T5 | 0.168 e | 0.157 g | 0.16 | 0.179 ef | 0.187 g | 0.18 |
| T6 | 0.113 f | 0.104 i | 0.10 | 0.165 f | 0.152 h | 0.15 |
| T7 | 0.181 de | 0.168 g | 0.17 | 0.222 bcd | 0.196 f | 0.20 |
| T8 | 0.234 ab | 0.218 d | 0.22 | 0.253 bc | 0.149 h | 0.20 |
| T9 | 0.226 bcd | 0.21 e | 0.21 | 0.195 def | 0.185 g | 0.19 |
| T10 | 0.322 a | 0.307 a | 0.31 | 0.373 a | 0.355 a | 0.36 |
| T11 | 0.094 f | 0.089 j | 0.09 | 0.158 f | 0.136 i | 0.147 |

Values followed by different letters in a column were significantly different (P<0.005). Data are average of four replicates (S- Sensitive Variety, T-Tolerant Variety).

**T_1_-** Seeds inoculated with P_1_, **T_2_-** Seeds inoculated with P_1_ + Plants Sprayed with SA and Put, **T_3_-** Seeds inoculated with P_2_ and P_3_, **T_4_-** Seeds inoculated with P_2_ and P_3_+ Plants Sprayed with SA and Put, **T_5_-** Seeds inoculated with P_1_, P_2_ and P_3_, **T_6_-** Seeds inoculated with P_1_, P_2_ and P_3_ + Plants Sprayed with SA and Put, **T_7_-** Plants sprayed with SA, **T_8_-** Plants sprayed with Put, **T_9_-** Plants sprayed with SA and Put, **T_10_-** Untreated control plants grown in sandy soil, **T_11_-** Irrigated control plants.
