# Supplementary material for: Impacts of plant growth promoters and plant growth regulators on rainfed agriculture
Source: PLoS One. 2020 Apr 9;15(4):e0231426. doi: 10.1371/journal.pone.0231426 (PMC7145150; doi:10.1371/journal.pone.0231426)
Supplement: S3 Table — (DOCX) [file pone.0231426.s003.docx]

**S3 Table. Effect of PGPR inoculation and PGR treatment alone or in combination on leaf protein content (ug/g) of chickpea grown in sandy soil.**

| **Treatments** | **2014-15 (S)** | **2015-16 (S)** | **Mean** | **2014-15**  **(T)** | **2015-16 (T)** | **Mean** |
| --- | --- | --- | --- | --- | --- | --- |
| T1 | 1.69 a | 1.76 a | 1.72 | 1.48 b | 1.50 c | 1.49 |
| T2 | 1.34 b | 1.39 cd | 1.36 | 1.20 cd | 1.29 d | 1.24 |
| T3 | 1.17 c | 1.24 e | 1.20 | 1.26 c | 1.23 de | 1.24 |
| T4 | 0.97 d | 1.02 f | 0.99 | 1.09 cd | 1.10 f | 1.09 |
| T5 | 1.69 a | 1.73 ab | 1.71 | 1.84 a | 1.87 a | 1.85 |
| T6 | 1.61 a | 1.71 b | 1.66 | 1.50 b | 1.69 b | 1.59 |
| T7 | 1.40 b | 1.44 c | 1.42 | 1.07 cd | 1.14 ef | 1.10 |
| T8 | 1.37 b | 1.37 cd | 1.37 | 1.51 b | 1.50 c | 1.50 |
| T9 | 1.58 a | 1.73 ab | 1.65 | 1.66 ab | 1.81 a | 1.73 |
| T10 | 1.06 cd | 1.09 f | 1.07 | 1.23 c | 1.26 d | 1.24 |
| T11 | 1.29 b | 1.31 de | 1.3 | 1.01 d | 1.12 f | 1.06 |

Values followed by different letters in a column were significantly different (P<0.005). Data are average of four replicates (S- Sensitive Variety, T-Tolerant Variety).
