# Supplementary material for: Impacts of plant growth promoters and plant growth regulators on rainfed agriculture
Source: PLoS One. 2020 Apr 9;15(4):e0231426. doi: 10.1371/journal.pone.0231426 (PMC7145150; doi:10.1371/journal.pone.0231426)
Supplement: S4 Table — (DOCX) [file pone.0231426.s004.docx]

**S4 Table. Effect of PGPR inoculation and PGR treatment alone or in combination on lipid peroxidation (nmol/g fwt.) of chickpea grown in sandy soil.**

| **Treatments** | **2014-15 (S)** | **2015-16 (S)** | **Mean** | **2014-15**  **(T)** | **2015-16 (T)** | **Mean** |
| --- | --- | --- | --- | --- | --- | --- |
| T1 | 0.105 b | 0.109 cd | 0.10 | 0.052 cd | 0.083 d | 0.06 |
| T2 | 0.060 c | 0.064 e | 0.06 | 0.045 d | 0.049 e | 0.04 |
| T3 | 0.124 b | 0.136 b | 0.13 | 0.072 c | 0.093 c | 0.06 |
| T4 | 0.104 b | 0.119 bcd | 0.11 | 0.052 cd | 0.051 e | 0.05 |
| T5 | 0.056 c | 0.049 ef | 0.05 | 0.040 d | 0.035 f | 0.03 |
| T6 | 0.030 c | 0.023 g | 0.02 | 0.030 d | 0.024 g | 0.02 |
| T7 | 0.103 b | 0.105 d | 0.10 | 0.104 b | 0.109 b | 0.10 |
| T8 | 0.125 b | 0.126 bc | 0.12 | 0.111 ab | 0.110 b | 0.11 |
| T9 | 0.042 c | 0.040 fg | 0.04 | 0.038 d | 0.037 f | 0.05 |
| T10 | 0.165 a | 0.170 a | 0.16 | 0.130 a | 0.135 a | 0.13 |
| T11 | 0.060 c | 0.063 e | 0.06 | 0.050 cd | 0.053 e | 0.07 |

Values followed by different letters in a column were significantly different (P<0.005). Data are average of four replicates (S- Sensitive Variety, T-Tolerant Variety).
