# Supplementary material for: Impacts of plant growth promoters and plant growth regulators on rainfed agriculture
Source: PLoS One. 2020 Apr 9;15(4):e0231426. doi: 10.1371/journal.pone.0231426 (PMC7145150; doi:10.1371/journal.pone.0231426)
Supplement: S5 Table — (DOCX) [file pone.0231426.s005.docx]

**S5 Table. Effect of PGPR inoculation and PGR treatment alone or in combination on leaf sugar content (mg/g) of chickpea grown in sandy soil.**

| **Treatments** | **2014-15 (S)** | **2015-16 (S)** | **Mean** | **2014-15**  **(T)** | **2015-16**  **(T)** | **Mean** |
| --- | --- | --- | --- | --- | --- | --- |
| T1 | 1.79 de | 1.82 ef | 2.7 | 1.98 bc | 2.04 cd | 3 |
| T2 | 2.16 bc | 2.19 c | 3.25 | 2.30 a | 2.50 a | 3.5 |
| T3 | 1.92 d | 2 d | 2.92 | 2.03 b | 2.16 c | 3.11 |
| T4 | 2.23 bc | 2.34 b | 3.4 | 1.73 d | 1.81 f | 2.63 |
| T5 | 2.41 a | 2.46 a | 3.64 | 2.26 a | 2.31 b | 3.41 |
| T6 | 2.28 ab | 2.34 b | 3.45 | 2.26 a | 2.32 b | 3.42 |
| T7 | 2.10 c | 2.15 c | 3.17 | 1.83 cd | 1.95 de | 2.80 |
| T8 | 1.66 e | 1.76 f | 2.54 | 1.81 cd | 1.84 ef | 2.73 |
| T9 | 1.84 d | 1.85 ef | 2.76 | 1.84 cd | 1.87 ef | 2.77 |
| T10 | 1.19 f | 1.19 g | 1.78 | 1.30 e | 1.36 g | 1.98 |
| T11 | 1.85 d | 1.90 df | 2.8 | 1.98 bc | 2.02 d | 2.99 |

Values followed by different letters in a column were significantly different (P<0.005). Data are average of four replicates (S- Sensitive Variety, T-Tolerant Variety).
