# Supplementary material for: Impacts of plant growth promoters and plant growth regulators on rainfed agriculture
Source: PLoS One. 2020 Apr 9;15(4):e0231426. doi: 10.1371/journal.pone.0231426 (PMC7145150; doi:10.1371/journal.pone.0231426)
Supplement: S6 Table — (DOCX) [file pone.0231426.s006.docx]

**S6 Table. Effect of PGPR inoculation and PGR treatment alone or in combination on root sugar content (mg/g) of chickpea grown in sandy soil.**

| **Treatments** | **2014-15 (S)** | **2015-16 (S)** | **Mean** | **2014-15 (T)** | **2015-16 (T)** | **Mean** |
| --- | --- | --- | --- | --- | --- | --- |
| T1 | 1.84 bc | 1.79 e | 2.73 | 2.07 a | 2.13 a | 3.13 |
| T2 | 1.91 bc | 1.91 d | 2.86 | 1.99 a | 2.17 a | 3.07 |
| T3 | 2 ab | 2.20 ab | 3.1 | 1.87 ab | 1.88 b | 2.81 |
| T4 | 1.88 bc | 1.91 d | 2.83 | 1.88 ab | 1.95 b | 2.85 |
| T5 | 2.19 a | 2.27 ab | 3.32 | 2.09 a | 2.19 a | 3.18 |
| T6 | 2.02 ab | 2.29 a | 3.16 | 2.11 a | 2.17 a | 3.19 |
| T7 | 2.01 ab | 2.17 bc | 3.09 | 1.49 c | 1.62 c | 2.3 |
| T8 | 1.74 cd | 1.80 de | 2.64 | 1.85 ab | 1.89 b | 2.79 |
| T9 | 1.94 b | 2.06 c | 2.97 | 2 a | 2.25 a | 3.12 |
| T10 | 0.984 e | 0.94 g | 1.45 | 1.21 d | 1.13 d | 1.77 |
| T11 | 1.57 d | 1.60 f | 2.37 | 1.61 bc | 1.63 c | 2.42 |

Values followed by different letters in a column were significantly different (P<0.005). Data are average of four replicates (S- Sensitive Variety, T-Tolerant Variety).
