# Supplementary material for: Impacts of plant growth promoters and plant growth regulators on rainfed agriculture
Source: PLoS One. 2020 Apr 9;15(4):e0231426. doi: 10.1371/journal.pone.0231426 (PMC7145150; doi:10.1371/journal.pone.0231426)
Supplement: S7 Table — (DOCX) [file pone.0231426.s007.docx]

**S7 Table. Effect of PGPR inoculation and PGR treatment alone or in combination on leaf phenolic content (mM GA eq/g FW) of chickpea grown in sandy soil.**

| **Treatments** | **2014-15 (S)** | **2015-16 (S)** | **Mean** | **2014-15**  **(T)** | **2015-16 (T)** | **Mean** |
| --- | --- | --- | --- | --- | --- | --- |
| T1 | 2.01 de | 2.11 f | 3.06 | 2.04 g | 2.18 h | 3.13 |
| T2 | 2.48 cd | 2.54 e | 3.75 | 3.22 de | 3.28 e | 4.86 |
| T3 | 2.45 cd | 2.46 e | 3.68 | 3.56 bcd | 3.60 cd | 5.36 |
| T4 | 2.98 c | 3.10 c | 4.53 | 4.03 ab | 4.20 b | 6.13 |
| T5 | 3.72 b | 3.79 b | 5.61 | 4.33 a | 4.48 a | 6.57 |
| T6 | 4.52 a | 4.70 a | 6.87 | 4.43 a | 4.47 a | 6.66 |
| T7 | 3.75 b | 3.73 b | 5.61 | 3.69 bc | 3.72 c | 5.55 |
| T8 | 2.84 c | 2.86 d | 4.27 | 2.66 f | 2.73 g | 4.02 |
| T9 | 4.47 a | 4.61 a | 6.77 | 3.47 cd | 3.54 d | 5.24 |
| T10 | 1.53 e | 1.55 g | 2.30 | 1.96 g | 2 i | 2.96 |
| T11 | 2.86 c | 2.90 d | 4.31 | 2.97 ef | 3.03 f | 4.48 |

Values followed by different letters in a column were significantly different (P<0.005). Data are average of four replicates (S- Sensitive Variety, T-Tolerant Variety).
