# Supplementary material for: Impacts of plant growth promoters and plant growth regulators on rainfed agriculture
Source: PLoS One. 2020 Apr 9;15(4):e0231426. doi: 10.1371/journal.pone.0231426 (PMC7145150; doi:10.1371/journal.pone.0231426)
Supplement: S8 Table — (DOCX) [file pone.0231426.s008.docx]

**S8 Table. Effect of PGPR inoculation and PGR treatment alone or in combination on catalase activity (units/g fwt.) in the leaves of chickpea grown in sandy soil.**

| **Treatments** | **2014-15 (S)** | **2015-16 (S)** | **Mean** | **2014-15 (T)** | **2015-16 (T)** | | **Mean** |
| --- | --- | --- | --- | --- | --- | --- | --- |
| T1 | 0.541 b | 0.572 b | 0.55 | 0.532 b | 0.555 b | 0.54 | |
| T2 | 0.414 c | 0.481 c | 0.44 | 0.421cd | 0.498 d | 0.45 | |
| T3 | 0.344 de | 0.356 e | 0.35 | 0.399 cde | 0.396 f | 0.38 | |
| T4 | 0.252 f | 0.241 g | 0.24 | 0.368 e | 0.371 g | 0.36 | |
| T5 | 0.333 e | 0.345 e | 0.33 | 0.38 de | 0.4 f | 0.39 | |
| T6 | 0.259 f | 0.277 f | 0.26 | 0.348 e | 0.38 g | 0.36 | |
| T7 | 0.434 c | 0.459 d | 0.44 | 0.38 de | 0.369 g | 0.37 | |
| T8 | 0.55 b | 0.559 b | 0.55 | 0.535 b | 0.527 c | 0.53 | |
| T9 | 0.388 cd | 0.359 e | 0.37 | 0.433 b | 0.419 e | 0.42 | |
| T10 | 0.669 a | 0.687 a | 0.67 | 0.73 a | 0.575 a | 0.65 | |
| T11 | 0.125 g | 0.14 h | 0.13 | 0.155 f | 0.17 h | 0.16 | |

Values followed by different letters in a column were significantly different (P<0.005). Data are average of four replicates (S- Sensitive Variety, T-Tolerant Variety).
