# Supplementary material for: Impacts of plant growth promoters and plant growth regulators on rainfed agriculture
Source: PLoS One. 2020 Apr 9;15(4):e0231426. doi: 10.1371/journal.pone.0231426 (PMC7145150; doi:10.1371/journal.pone.0231426)
Supplement: S9 Table — (DOCX) [file pone.0231426.s009.docx]

**S9 Table. Effect of PGPR inoculation and PGR treatment alone or in combination on ascorbate peroxidase (APOX) activity (units/g fwt.) in the leaves of chickpea grown in sandy soil.**

| **Treatments** | **2014-15 (S)** | **2015-16 (S)** | **Mean** | **2014-15 (T)** | **2015-16 (T)** | **Mean** |
| --- | --- | --- | --- | --- | --- | --- |
| T1 | 0.237 c | 0.254 d | 0.24 | 0.301 c | 0.319 d | 0.31 |
| T2 | 0.153 e | 0.165 f | 0.15 | 0.281 cd | 0.308 e | 0.29 |
| T3 | 0.399 b | 0.406 b | 0.40 | 0.381 b | 0.382 b | 0.38 |
| T4 | 0.383 b | 0.38 c | 0.38 | 0.365 b | 0.357 c | 0.36 |
| T5 | 0.208 d | 0.224 e | 0.21 | 0.249 d | 0.265 f | 0.25 |
| T6 | 0.128 f | 0.115 g | 0.12 | 0.182 e | 0.189 g | 0.18 |
| T7 | 0.086 g | 0.102 h | 0.094 | 0.108 f | 0.13 h | 0.11 |
| T8 | 0.1 g | 0.116 g | 0.108 | 0.109 f | 0.126 hi | 0.11 |
| T9 | 0.085 g | 0.101 h | 0.093 | 0.093 f | 0.115 i | 0.10 |
| T10 | 0.434 a | 0.448 a | 0.44 | 0.567 a | 0.574 a | 0.57 |
| T11 | 0.032 h | 0.042 i | 0.03 | 0.045 g | 0.06 j | 0.05 |

Values followed by different letters in a column were significantly different (P<0.005). Data are average of four replicates (S- Sensitive Variety, T-Tolerant Variety).
