# Supplementary material for: Impacts of plant growth promoters and plant growth regulators on rainfed agriculture
Source: PLoS One. 2020 Apr 9;15(4):e0231426. doi: 10.1371/journal.pone.0231426 (PMC7145150; doi:10.1371/journal.pone.0231426)
Supplement: S10 Table — (DOCX) [file pone.0231426.s010.docx]

**S10 Table. Effect of PGPR inoculation and PGR treatment alone or in combination on peroxidase activity (units/g fwt.) in the leaves of chickpea grown in sandy soil.**

| **Treatments** | **2014-15 (S)** | **2015-16 (S)** | **Mean** | **2014-15 (T)** | **2015-16 (T)** | **Mean** |
| --- | --- | --- | --- | --- | --- | --- |
| T1 | 0.246 ac | 0.253 b | 0.24 | 0.304 b | 0.324 b | 0.31 |
| T2 | 0.215 d | 0.225 cd | 0.22 | 0.272 c | 0.266 e | 0.26 |
| T3 | 0.209 d | 0.261 b | 0.23 | 0.258 c | 0.210 f | 0.23 |
| T4 | 0.167 e | 0.178 e | 0.17 | 0.269 c | 0.283 cd | 0.27 |
| T5 | 0.143 ef | 0.164 f | 0.15 | 0.184 d | 0.190 g | 0.18 |
| T6 | 0.115 f | 0.122 g | 0.11 | 0.159 d | 0.173 h | 0.16 |
| T7 | 0.257 b | 0.260 b | 0.25 | 0.278 bc | 0.282 d | 0.28 |
| T8 | 0.22 cd | 0.230 c | 0.22 | 0.288 bc | 0.296 c | 0.29 |
| T9 | 0.213 d | 0.216 d | 0.21 | 0.264 c | 0.274 de | 0.26 |
| T10 | 0.287 a | 0.281 a | 0.28 | 0.339 a | 0.355 a | 0.34 |
| T11 | 0.082 g | 0.084 h | 0.08 | 0.104 e | 0.111 i | 0.10 |

Values followed by different letters in a column were significantly different (P<0.005). Data are average of four replicates (S- Sensitive Variety, T-Tolerant Variety).
