# Supplementary material for: Impacts of plant growth promoters and plant growth regulators on rainfed agriculture
Source: PLoS One. 2020 Apr 9;15(4):e0231426. doi: 10.1371/journal.pone.0231426 (PMC7145150; doi:10.1371/journal.pone.0231426)
Supplement: S11 Table — (DOCX) [file pone.0231426.s011.docx]

S11 Table. Effect of PGPR inoculation and PGR treatment alone or in combination on superoxide dismutase activity (units/g fwt.) in the leaves of chickpea grown in sandy soil.

| **Treatments** | **2014-15 (S)** | **2015-16 (S)** | **Mean** | **2014-15 (T)** | **2015-16 (T)** | **Mean** |
| --- | --- | --- | --- | --- | --- | --- |
| T1 | 0.632 b | 0.648 b | 0.64 | 0.574 b | 0.585 b | 0.872 |
| T2 | 0.591 bc | 0.604 c | 0.59 | 0.541 bc | 0.555 c | 0.57 |
| T3 | 0.462 d | 0.474 e | 0.46 | 0.432 de | 0.435 e | 0.43 |
| T4 | 0.417 de | 0.424 f | 0.42 | 0.389 ef | 0.401 f | 0.39 |
| T5 | 0.375 ef | 0.386 g | 0.38 | 0.383 ef | 0.389 g | 0.38 |
| T6 | 0.324 g | 0.331 i | 0.32 | 0.338 f | 0.344 h | 0.34 |
| T7 | 0.240 h | 0.241 k | 0.24 | 0.426 de | 0.436 e | 0.43 |
| T8 | 0.573 c | 0.579 d | 0.57 | 0.491 cd | 0.494 d | 0.49 |
| T9 | 0.352 fg | 0.361 h | 0.35 | 0.488 cd | 0.493 d | 0.49 |
| T10 | 0.826 a | 0.835 a | 0.83 | 0.974 a | 0.986 a | 0.98 |
| T11 | 0.263 h | 0.266 j | 0.26 | 0.323 f | 0.326 i | 0.32 |

Values followed by different letters in a column were significantly different (P<0.005). Data are average of four replicates (S- Sensitive Variety, T-Tolerant Variety).
