# Supplementary material for: Impacts of plant growth promoters and plant growth regulators on rainfed agriculture
Source: PLoS One. 2020 Apr 9;15(4):e0231426. doi: 10.1371/journal.pone.0231426 (PMC7145150; doi:10.1371/journal.pone.0231426)
Supplement: S12 Table — (DOCX) [file pone.0231426.s012.docx]

**S12 Table. Effect of PGPR inoculation and PGR treatment alone or in combination on relative water content (%) of chickpea grown in sandy soil.**

| **Treatments** | **2014-15 (S)** | **2015-16 (S)** | **Mean** | **2014-15 (T)** | **2015-16 (T)** | **Mean** |
| --- | --- | --- | --- | --- | --- | --- |
| T1 | 22.5 f | 23 g | 34 | 37.7 e | 39.3 e | 57.35 |
| T2 | 26.7 e | 27.1 f | 40.25 | 47.8 c | 49.7 c | 72.65 |
| T3 | 13.9 h | 14 i | 20.9 | 31.1 f | 28.5 f | 45.35 |
| T4 | 18.4 g | 20 h | 28.4 | 40.1 e | 37.2 e | 58.7 |
| T5 | 28.7 e | 30.3 e | 43.85 | 44.3 d | 47.2 cd | 67.9 |
| T6 | 45.5 b | 47.9 b | 96.45 | 56.9 b | 57.6 b | 85.7 |
| T7 | 32.3 d | 34.6 d | 49.6 | 45.9 cd | 45.4 d | 68.6 |
| T8 | 25.9 e | 27.6 f | 39.7 | 37.4 e | 37.8 e | 56.3 |
| T9 | 41.4 c | 44.1 c | 63.45 | 54.8 b | 56 b | 82.8 |
| T10 | 9.9 i | 10.6 j | 15.2 | 24.9 g | 25.1 g | 37.45 |
| T11 | 75.9 a | 76.6 a | 114.2 | 78.6 a | 80.5 a | 118.85 |

Values followed by different letters in a column were significantly different (P<0.005 Data are average of four replicates (S- Sensitive Variety, T-Tolerant Variety).
