# Supplementary material for: Impacts of plant growth promoters and plant growth regulators on rainfed agriculture
Source: PLoS One. 2020 Apr 9;15(4):e0231426. doi: 10.1371/journal.pone.0231426 (PMC7145150; doi:10.1371/journal.pone.0231426)
Supplement: S13 Table — (DOCX) [file pone.0231426.s013.docx]

**S13 Table. Effect of PGPR inoculation and PGR treatment alone or in combination on shoot fresh weight (g) of chickpea grown in sandy soil.**

| **Treatments** | **2014-15 (S)** | **2015-16 (S)** | **Mean** | **2014-15 (T)** | **2015-16 (T)** | | **Mean** |
| --- | --- | --- | --- | --- | --- | --- | --- |
| T1 | 18 cd | 22 cd | 29 | 20.9 e | 20.8 d | 31.3 | |
| T2 | 19.3 c | 20.7 de | 29.65 | 22.7 d | 23.1 d | 34.25 | |
| T3 | 15.7 e | 16.1 f | 23.75 | 18.1 f | 21.1 d | 28.65 | |
| T4 | 16.9 de | 19.7 de | 26.75 | 20.5 e | 22.7 d | 31.85 | |
| T5 | 22.7 b | 23.9 bc | 34.65 | 26.4 c | 26.6 c | 39.7 | |
| T6 | 24 b | 26.3 b | 37.15 | 29.8 b | 30.7 b | 45.15 | |
| T7 | 17.4 d | 18.5 ef | 26.65 | 14.7 g | 15 e | 22.2 | |
| T8 | 10 g | 9.8 g | 14.9 | 10.9 h | 9.1 fg | 15.45 | |
| T9 | 11.7 f | 10.3 g | 16.85 | 11.8 h | 11.1 f | 17.35 | |
| T10 | 4.5 h | 5.4 h | 7.2 | 7.4 i | 7.8 g | 11.3 | |
| T11 | 33.8 a | 33.7 a | 50.65 | 39.1 a | 39.9 a | 59.05 | |

Values followed by different letters in a column were significantly different (P<0.005). Data are average of four replicates (S- Sensitive Variety, T-Tolerant Variety).
