# Supplementary material for: Impacts of plant growth promoters and plant growth regulators on rainfed agriculture
Source: PLoS One. 2020 Apr 9;15(4):e0231426. doi: 10.1371/journal.pone.0231426 (PMC7145150; doi:10.1371/journal.pone.0231426)
Supplement: S14 Table — (DOCX) [file pone.0231426.s014.docx]

**S14 Table. Effect of PGPR inoculation and PGR treatment alone or in combination on shoot dry weight (g) of chickpea grown in sandy soil.**

| **Treatments** | **2014-15 (S)** | **2015-16 (S)** | **Mean** | **2014-15 (T)** | **2015-16 (T)** | **Mean** |
| --- | --- | --- | --- | --- | --- | --- |
| T1 | 10.3 b | 11.4 ab | 16 | 12.1 b | 12.9 ab | 18.55 |
| T2 | 10 bc | 10.6 ab | 15.3 | 12.9 a | 14 a | 19.9 |
| T3 | 7.7 e | 7.9 cd | 11.65 | 9 e | 9.8 bcd | 13.9 |
| T4 | 8.7 d | 9.1 bc | 13.25 | 10.1 d | 11.4 ab | 15.8 |
| T5 | 11.3 a | 11.9 a | 17.25 | 8.9 e | 10 bc | 13.9 |
| T6 | 11.9 a | 12.1 a | 17.95 | 11.2 c | 12.3 ab | 17.35 |
| T7 | 6.3 f | 6.8 cde | 9.7 | 5 g | 5.9 ef | 7.95 |
| T8 | 4 g | 5.1 ef | 6.55 | 5 g | 6.1 ef | 8.05 |
| T9 | 5.8 f | 5.8 de | 8.7 | 5.5 g | 6.8 def | 8.9 |
| T10 | 2.7 h | 3 f | 4.2 | 3.4 h | 4.2 f | 5.5 |
| T11 | 9.3 cd | 11 ab | 14.8 | 7.4 f | 7.7 cde | 11.25 |

Values followed by different letters in a column were significantly different (P<0.005). Data are average of four replicates (S- Sensitive Variety, T-Tolerant Variety).
