# Supplementary material for: Impacts of plant growth promoters and plant growth regulators on rainfed agriculture
Source: PLoS One. 2020 Apr 9;15(4):e0231426. doi: 10.1371/journal.pone.0231426 (PMC7145150; doi:10.1371/journal.pone.0231426)
Supplement: S15 Table — (DOCX) [file pone.0231426.s015.docx]

**S15 Table. Effect of PGPR inoculation and PGR treatment alone or in combination on root fresh weight (g) of grown in sandy soil.**

| **Treatments** | **2014-15 (S)** | **2015-16 (S)** | **Mean** | **2014-15 (T)** | **2015-16 (T)** | **Mean** |
| --- | --- | --- | --- | --- | --- | --- |
| T1 | 4.88 e | 5.11 f | 7.43 | 5.27 e | 5.36 e | 7.95 |
| T2 | 5.69 d | 5.72 e | 8.56 | 5.84 d | 5.95 d | 8.81 |
| T3 | 4.12 g | 4.27 h | 6.25 | 4.21 h | 4.35 h | 6.38 |
| T4 | 4.48 f | 4.56 g | 6.76 | 4.48 g | 4.59 g | 6.77 |
| T5 | 6.35 c | 6.4 c | 9.55 | 6.11 c | 6.34 c | 9.28 |
| T6 | 7.55 b | 7.7 b | 11.4 | 7.4 b | 7.72 b | 11.26 |
| T7 | 5.9 d | 6.07 d | 8.93 | 5.41 e | 5.49 e | 8.15 |
| T8 | 4.52 f | 4.56 g | 6.8 | 4.87 f | 4.88 f | 7.31 |
| T9 | 5.7 d | 5.81 e | 8.60 | 5.68 d | 5.83 d | 8.59 |
| T10 | 3.52 h | 3.54 i | 5.29 | 3.98 i | 4.07 i | 6.01 |
| T11 | 7.98 a | 8.09 a | 12.02 | 7.7 a | 7.92 a | 11.66 |

Values followed by different letters in a column were significantly different (P<0.005). Data are average of four replicates (S- Sensitive Variety, T-Tolerant Variety).
