# Supplementary material for: Impacts of plant growth promoters and plant growth regulators on rainfed agriculture
Source: PLoS One. 2020 Apr 9;15(4):e0231426. doi: 10.1371/journal.pone.0231426 (PMC7145150; doi:10.1371/journal.pone.0231426)
Supplement: S16 Table — (DOCX) [file pone.0231426.s016.docx]

**S16 Table. Effect of PGPR inoculation and PGR treatment alone or in combination on root dry weight (g) of chickpea grown in sandy soil.**

| **Treatments** | **2014-15 (S)** | **2015-16 (S)** | **Mean** | **2014-15 (T)** | **2015-16 (T)** | **Mean** |
| --- | --- | --- | --- | --- | --- | --- |
| T1 | 1.35 e | 1.39 ef | 2.05 | 1.53 e | 1.64 de | 2.35 |
| T2 | 1.54 cd | 1.64 d | 2.36 | 1.74 bc | 1.85 bc | 2.66 |
| T3 | 1.62 c | 1.71 d | 2.47 | 1.66 d | 1.76 cd | 2.54 |
| T4 | 1.64 c | 1.74 d | 2.51 | 1.80 b | 1.92 b | 2.76 |
| T5 | 1.89 b | 1.90 c | 2.84 | 1.69 cd | 1.85 bc | 2.61 |
| T6 | 2.29 a | 2.35 a | 3.46 | 2.11 a | 2.18 a | 3.2 |
| T7 | 1.37 e | 1.44 ef | 2.09 | 1.25 f | 1.13 f | 1.81 |
| T8 | 1.22 f | 1.33 f | 1.88 | 1.45 e | 1.56 e | 2.23 |
| T9 | 1.44 de | 1.50 e | 2.19 | 1.47 e | 1.58 e | 2.26 |
| T10 | 0.72 g | 0.85 g | 1.14 | 0.91 g | 1.06 f | 1.44 |
| T11 | 2.22 a | 2.22 b | 3.33 | 2.19 a | 2.1 a | 3.24 |

Values followed by different letters in a column were significantly different (P<0.005). Data are average of four replicates (S- Sensitive Variety, T-Tolerant Variety).
