# Supplementary material for: Impacts of plant growth promoters and plant growth regulators on rainfed agriculture
Source: PLoS One. 2020 Apr 9;15(4):e0231426. doi: 10.1371/journal.pone.0231426 (PMC7145150; doi:10.1371/journal.pone.0231426)
Supplement: S17 Table — (DOCX) [file pone.0231426.s017.docx]

**S17 Table. Effect of PGPR inoculation and PGR treatment alone or in combination on number of nodules per plant of chickpea grown in sandy soil.**

| **Treatments** | **2014-15 (S)** | **2015-16 (S)** | **Mean** | **2014-15 (T)** | **2015-16 (T)** | **Mean** |
| --- | --- | --- | --- | --- | --- | --- |
| T1 | 38.2 b | 40.7 b | 58.55 | 29.5 b | 28.5 bc | 43.75 |
| T2 | 28.5 c | 32.7 d | 44.85 | 20.5 f | 20.2 f | 30.6 |
| T3 | 22 d | 23 f | 33.5 | 26.2 bcd | 31.2 b | 41.8 |
| T4 | 19.2 de | 21.7 f | 29.85 | 21.5 ef | 21.7 ef | 32.35 |
| T5 | 42.7 a | 46.2 a | 65.8 | 35.7 a | 37.7 a | 54.55 |
| T6 | 35.2 b | 36.7 c | 53.55 | 27.7 bc | 27.7 cd | 41.55 |
| T7 | 4.2 g | 4.2 i | 6.3 | 7.7 i | 9 h | 12.2 |
| T8 | 27.2 c | 26.7 e | 40.55 | 25 cde | 23.5 e | 36.75 |
| T9 | 16.2 e | 17.7 g | 25.05 | 16.5 g | 19 f | 26 |
| T10 | 9.5 f | 9.5 h | 14.25 | 12.7 h | 13.5 g | 19.45 |
| T11 | 20.2 d | 22.2 f | 31.3 | 23.5 def | 24.7 de | 35.85 |

Values followed by different letters in a column were significantly different (P<0.005). Data are average of four replicates (S- Sensitive Variety, T-Tolerant Variety).
