# Supplementary material for: Impacts of plant growth promoters and plant growth regulators on rainfed agriculture
Source: PLoS One. 2020 Apr 9;15(4):e0231426. doi: 10.1371/journal.pone.0231426 (PMC7145150; doi:10.1371/journal.pone.0231426)
Supplement: S18 Table — (DOCX) [file pone.0231426.s018.docx]

**S18 Table. Effect of PGPR inoculation and PGR treatment alone or in combination on number of pods per plant of chickpea grown in sandy soil.**

| **Treatments** | **2014-15 (S)** | **2015-16 (S)** | **Mean** | **2014-15 (T)** | **2015-16 (T)** | **Mean** |
| --- | --- | --- | --- | --- | --- | --- |
| T1 | 42.5 e | 37.5 e | 61.25 | 49 e | 52.7 e | 75.35 |
| T2 | 27.7 fg | 28 fg | 41.7 | 43.5 e | 48.2 e | 67.6 |
| T3 | 30.7 e | 32.2 ef | 46.8 | 34.5 f | 40.2 f | 54.6 |
| T4 | 46.7 de | 51.5 d | 72.45 | 31.7 f | 34.2 g | 48.8 |
| T5 | 71 c | 75 c | 108.5 | 78.7 c | 83.2 c | 120.3 |
| T6 | 82.7 b | 84.7 b | 125.05 | 90.2 b | 87.2 b | 133.8 |
| T7 | 21.7 g | 24 g | 33.7 | 29.5 f | 29.2 g | 44.1 |
| T8 | 44.2 de | 50.7 d | 69.55 | 49.5 e | 52 e | 75.5 |
| T9 | 50.5 d | 52.7 d | 76.85 | 57.7 d | 60.2 d | 87.8 |
| T10 | 10 h | 11.7 h | 15.85 | 18.7 g | 17.7 h | 27.55 |
| T11 | 113.7 a | 125.5 a | 176.45 | 133.7 a | 112.5 a | 189.95 |

Values followed by different letters in a column were significantly different (P<0.005). Data are average of four replicates (S- Sensitive Variety, T-Tolerant Variety).
