# Supplementary material for: Impacts of plant growth promoters and plant growth regulators on rainfed agriculture
Source: PLoS One. 2020 Apr 9;15(4):e0231426. doi: 10.1371/journal.pone.0231426 (PMC7145150; doi:10.1371/journal.pone.0231426)
Supplement: S19 Table — (DOCX) [file pone.0231426.s019.docx]

**S19 Table. Effect of PGPR inoculation and PGR treatment alone or in combination on 100-pod weight (g) of chickpea grown in sandy soil.**

| **Treatments** | **2014-15 (S)** | **2015-16 (S)** | **Mean** | **2014-15 (T)** | **2015-16 (T)** | **Mean** |
| --- | --- | --- | --- | --- | --- | --- |
| T1 | 24.9 f | 26.1 e | **25.5** | 36.1 de | 33.4 d | **34.7** |
| T2 | 32 e | 32.5 d | **32.2** | 40.6 c | 38.3 c | **39.4** |
| T3 | 30.3 e | 33.7 d | 32 | 34.6 e | 36.2 c | 35.4 |
| T4 | 34.6 d | 35.1 cd | 34.8 | 41.2 bc | 43.1 b | 42.1 |
| T5 | 34.2 d | 36.8 c | 35.5 | 37.4 d | 42.1 b | 39.7 |
| T6 | 45.9 ab | 46.9 a | 46.4 | 43.3 a | 46.8 a | 45 |
| T7 | 44.7 b | 47.6 a | 46.1 | 42.8 ab | 47 a | 44.9 |
| T8 | 23 g | 27.3 e | 25.1 | 29 f | 32 d | 30.5 |
| T9 | 36.8 c | 39.7 b | 38.2 | 41.8 abc | 43.5 b | 42.6 |
| T10 | 21.6 g | 20.4 f | 21 | 25.5 g | 23 e | 24.2 |
| T11 | 47.4 a | 48.3 a | 47.8 | 43.4 a | 47 b | 45.2 |

Values followed by different letters in a column were significantly different (P<0.005). Data are average of four replicates (S- Sensitive Variety, T-Tolerant Variety).
