# Supplementary material for: Impacts of plant growth promoters and plant growth regulators on rainfed agriculture
Source: PLoS One. 2020 Apr 9;15(4):e0231426. doi: 10.1371/journal.pone.0231426 (PMC7145150; doi:10.1371/journal.pone.0231426)
Supplement: S20 Table — (DOCX) [file pone.0231426.s020.docx]

**S20 Table. Effect of PGPR inoculation and PGR treatment alone or in combination on 100-grain weight (g) of chickpea grown in sandy soil.**

| **Treatments** | **2014-15 (S)** | **2015-16 (S)** | **Mean** | **2014-15 (T)** | **2015-16 (T)** | **Mean** |
| --- | --- | --- | --- | --- | --- | --- |
| T1 | 18.9 f | 20.7 c | 19.8 | 21.4 d | 22 b | 21.7 |
| T2 | 21 e | 22.4 bc | 21.7 | 23 c | 23.7 b | 23.3 |
| T3 | 21.7 de | 22 bc | 21.8 | 21.6 d | 22.6 b | 22.1 |
| T4 | 22.3 d | 22.9 bc | 22.6 | 23.1 c | 23.5 b | 23.3 |
| T5 | 22.7 d | 23.7 b | 23.2 | 21.8 d | 23.2 b | 22.5 |
| T6 | 26.1 b | 27.7 a | 26.9 | 26.2 a | 26.4 a | 26.3 |
| T7 | 28.1 a | 27.6 a | 27.8 | 25.9 a | 27.2 a | 26.5 |
| T8 | 17.1 g | 17.4 d | 17.2 | 18.2 e | 19.6 c | 18.9 |
| T9 | 24 c | 26.6 a | 25.3 | 24.8 | 26.7 a | 25.7 |
| T10 | 16.3 g | 15.8 d | 16 | 19 e | 18.7 c | 18.8 |
| T11 | 27.2 ab | 28.9 a | 28 | 24.1 b | 26.9 a | 25.5 |

Values followed by different letters in a column were significantly different (P<0.005). Data are average of four replicates (S- Sensitive Variety, T-Tolerant Variety).
