# Supplementary material for: Impacts of plant growth promoters and plant growth regulators on rainfed agriculture
Source: PLoS One. 2020 Apr 9;15(4):e0231426. doi: 10.1371/journal.pone.0231426 (PMC7145150; doi:10.1371/journal.pone.0231426)
Supplement: S21 Table — (DOCX) [file pone.0231426.s021.docx]

**S21 Table. Effect of PGPR inoculation and PGRs treatment alone or in combination on plant height (cm) of chickpea grown in sandy soil.**

| **Treatments** | **2014-15 (S)** | **2015-16 (S)** | **Mean** | **2014-15 (T)** | **2015-16 (T)** | **Mean** |
| --- | --- | --- | --- | --- | --- | --- |
| T1 | 40.1 d | 42.4 c | 41.2 | 42.8 c | 43.6 c | 43.2 |
| T2 | 37.6 e | 40 cd | 38.8 | 40.2 cd | 42.3 c | 41.2 |
| T3 | 33.1 f | 32.1 f | 32.6 | 35.8 e | 33.2 e | 34.5 |
| T4 | 36.6 e | 36.1 e | 36.3 | 38.7 de | 38.8 d | 38.7 |
| T5 | 44.8 b | 46 b | 45.4 | 51.5 b | 51.7 b | 51.6 |
| T6 | 41.9 c | 42.5 c | 42.2 | 40.8 cd | 43.4 c | 42.1 |
| T7 | 37.5 e | 38.8 de | 38.1 | 36 e | 36.1 de | 36 |
| T8 | 23.7 g | 23.7 g | 23.7 | 25.5 f | 26.2 f | 25.8 |
| T9 | 32.5 f | 33.3 f | 32.9 | 35.9 e | 36.7 d | 36.3 |
| T10 | 17.3 h | 17.7 h | 17.5 | 22.6 f | 22.5 g | 22.5 |
| T11 | 75.2 a | 75.1 a | 75.1 | 78.7 a | 79.6 a | 79.1 |

Values followed by different letters in a column were significantly different (P<0.005). Data are average of four replicates (S- Sensitive Variety, T-Tolerant Variety).
