# Supplementary material for: Impacts of plant growth promoters and plant growth regulators on rainfed agriculture
Source: PLoS One. 2020 Apr 9;15(4):e0231426. doi: 10.1371/journal.pone.0231426 (PMC7145150; doi:10.1371/journal.pone.0231426)
Supplement: S22 Table — (DOCX) [file pone.0231426.s022.docx]

**S22 Table. Effect of PGPR inoculation and PGR treatment alone or in combination on yield per 5-plants (g) of chickpea grown in sandy soil.**

| **Treatments** | **2014-15 (S)** | **2015-16 (S)** | **Mean** | **2014-15 (T)** | **2015-16 (T)** | **Mean** |
| --- | --- | --- | --- | --- | --- | --- |
| T1 | 25.7 f | 25.7 f | 25.7 | 25.3 f | 26.6 gh | 25.9 |
| T2 | 37.5 d | 39.2 d | 38.3 | 36.6 d | 36.7 e | 36.6 |
| T3 | 23.1 f | 26.2 f | 24.6 | 27.6 ef | 29.3 fg | 28.4 |
| T4 | 25.2 f | 29 f | 27.1 | 24.5 f | 25.5 h | 25 |
| T5 | 49.1 b | 49.9 c | 49.5 | 50.5 b | 51.4 b | 50.9 |
| T6 | 52.1 b | 55.3 b | 53.7 | 43.2 c | 47.7 c | 45.4 |
| T7 | 42.3 c | 40.9 d | 41.6 | 45.3 c | 43.7 d | 44.5 |
| T8 | 33.1 d | 34.7 e | 33.9 | 37 d | 41.1 d | 39 |
| T9 | 26.5 e | 28.2 f | 27.3 | 29.7 e | 31.7 f | 30.7 |
| T10 | 12.2 g | 12 g | 12.1 | 15.9 g | 16 i | 15.9 |
| T11 | 61.5 a | 64 a | 62.7 | 63.8 a | 65.7 a | 64.7 |

Values followed by different letters in a column were significantly different (P<0.005). Data are average of four replicates (S- Sensitive Variety, T-Tolerant Variety).
