# Supplementary material for: Impacts of plant growth promoters and plant growth regulators on rainfed agriculture
Source: PLoS One. 2020 Apr 9;15(4):e0231426. doi: 10.1371/journal.pone.0231426 (PMC7145150; doi:10.1371/journal.pone.0231426)
Supplement: S23 Table — (DOCX) [file pone.0231426.s023.docx]

**S23 Table. Effect of PGPR inoculation and PGR treatment alone or in combination on total biomass (g) of chickpea grown in sandy soil.**

| **Treatments** | **2014-15 (S)** | **2015-16 (S)** | **Mean** | **2014-15 (T)** | **2015-16 (T)** | **Mean** |
| --- | --- | --- | --- | --- | --- | --- |
| T1 | 48.5 f | 49.1 f | 48.8 | 49.8 cd | 50.9 f | 50.3 |
| T2 | 67.5 d | 60.3 e | 63.9 | 69 abc | 66.1 d | 67.5 |
| T3 | 53.6 ef | 53.8 f | 53.7 | 58.2 bcd | 60.6 e | 59.4 |
| T4 | 59.6 e | 61.8 e | 60.7 | 50.9 cd | 51.3 f | 51.1 |
| T5 | 85.9 b | 90.7 b | 88.3 | 89.8 a | 91.3 a | 90.5 |
| T6 | 82.6 b | 84.4 c | 83.5 | 81.6 ab | 84.7 b | 83.1 |
| T7 | 82.5 b | 82.9 c | 82.7 | 89.7 a | 89.9 a | 89.8 |
| T8 | 75.3 c | 70.5 d | 72.9 | 80.1 ab | 74.4 c | 77.2 |
| T9 | 57.1 e | 61.1 e | 59.1 | 69.8 abc | 71.7 c | 70.7 |
| T10 | 38.8 g | 38.2 g | 38.5 | 42 d | 41 g | 41.5 |
| T11 | 106.6 a | 108.3 a | 107.4 | 91 a | 92.7 a | 91.8 |

Values followed by different letters in a column were significantly different (P<0.005). Data are average of four replicates (S- Sensitive Variety, T-Tolerant Variety).
