# Supplementary material for: Impacts of plant growth promoters and plant growth regulators on rainfed agriculture
Source: PLoS One. 2020 Apr 9;15(4):e0231426. doi: 10.1371/journal.pone.0231426 (PMC7145150; doi:10.1371/journal.pone.0231426)
Supplement: S24 Table — (DOCX) [file pone.0231426.s024.docx]

**S24 Table. Effect of PGPR inoculation and PGR treatment alone or in combination on harvest index (%) of chickpea grown in sandy soil.**

| **Treatments** | **2014-15 (S)** | **2015-16 (S)** | **Mean** | **2014-15 (T)** | **2015-16 (T)** | **Mean** |
| --- | --- | --- | --- | --- | --- | --- |
| T1 | 0.52 cd | 0.53 bcd | 0.52 | 0.50 cd | 0.56 cd | 0.53 |
| T2 | 0.55 bc | 0.57 bc | 0.56 | 0.52 c | 0.58 bc | 0.55 |
| T3 | 0.42 f | 0.49 def | 0.45 | 0.47 ef | 0.50 de | 0.48 |
| T4 | 0.42 f | 0.45 f | 0.43 | 0.47 def | 0.53 cde | 0.5 |
| T5 | 0.56 b | 0.59 b | 0.57 | 0.56 b | 0.58 bc | 0.57 |
| T6 | 0.62 a | 0.65 a | 0.63 | 0.59 a | 0.64 ab | 0.61 |
| T7 | 0.51 d | 0.53 cde | 0.52 | 0.50 cde | 0.51 de | 0.50 |
| T8 | 0.44 ef | 0.45 f | 0.44 | 0.46 f | 0.47 ef | 0.46 |
| T9 | 0.46 e | 0.48 ef | 0.47 | 0.42 g | 0.47 ef | 0.44 |
| T10 | 0.31 g | 0.32 g | 0.31 | 0.37 h | 0.40 f | 0.38 |
| T11 | 0.63 a | 0.68 a | 0.65 | 0.61 a | 0.65 a | 0.63 |

Values followed by different letters in a column were significantly different (P<0.005). Data are average of four replicates (S- Sensitive Variety, T-Tolerant Variety).
